# koopmans: An Open-Source Package for Accurately and Efficiently Predicting Spectral Properties with Koopmans Functionals

**DOI:** 10.1021/acs.jctc.3c00652

**Published:** 2023-08-23

**Authors:** Edward B. Linscott, Nicola Colonna, Riccardo De Gennaro, Ngoc Linh Nguyen, Giovanni Borghi, Andrea Ferretti, Ismaila Dabo, Nicola Marzari

**Affiliations:** † Theory and Simulation of Materials (THEOS), École Polytechnique Fédérale de Lausanne, 1015 Lausanne, Switzerland; ‡ Laboratory for Neutron Scattering and Imaging, Paul Scherrer Institut, 5232 Villigen, Switzerland; ¶ National Centre for Computational Design and Discovery of Novel Materials (MARVEL), École Polytechnique Fédérale de Lausanne, 1015 Lausanne, Switzerland; § Faculty of Materials Science and Engineering, Phenikaa University, Hanoi 12116, Vietnam; ∥ A&A Green Phoenix Group JSC, Phenikaa Research and Technology Institute (PRATI), No. 167 Hoang Ngan, Trung Hoa, Cau Giay, Hanoi 11313, Vietnam; ⊥ Centro S3, CNR−Istituto Nanoscienze, 41125 Modena, Italy; # Department of Materials Science and Engineering, Materials Research Institute, and Institutes of Energy and the Environment, The Pennsylvania State University, University Park, Pennsylvania 16802, United States; @ Laboratory for Materials Simulations, Paul Scherrer Institut, 5232 Villigen, Switzerland

## Abstract

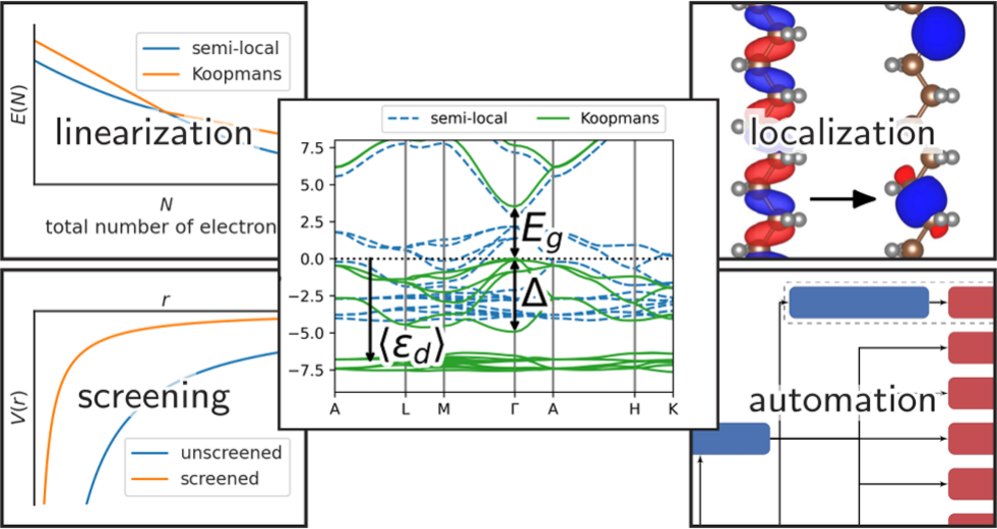

Over the past decade we have developed Koopmans functionals, a computationally efficient approach for predicting spectral properties with an orbital-density-dependent functional framework. These functionals impose a generalized piecewise linearity condition to the entire electronic manifold, ensuring that orbital energies match the corresponding electron removal/addition energy differences (in contrast to semilocal DFT, where a mismatch between the two lies at the heart of the band gap problem and, more generally, the unreliability of Kohn–Sham orbital energies). This strategy has proven to be very powerful, yielding molecular orbital energies and solid-state band structures with comparable accuracy to many-body perturbation theory but at greatly reduced computational cost while preserving a functional formulation. This paper reviews the theory of Koopmans functionals, discusses the algorithms necessary for their implementation, and introduces koopmans, an open-source package that contains all of the code and workflows needed to perform Koopmans functional calculations and obtain reliable spectral properties of molecules and materials.

## 1 Introduction

How can one accurately and efficiently predict spectral properties of molecules and materials *ab initio*? Currently, the most accurate and popular approaches to compute charged excitation energies are Green’s functions methods such as many-body perturbation theory (GW)^1,2^ or wave function methods such as quantum Monte Carlo^3^ and equation-of-motion coupled cluster.^4^ Although for the latter, calculations for the solid state (rather than for molecules) are far from routine. Of these approaches, GW is computationally the least expensive, scaling as , where *N* is the number of electrons in the system.

Despite ongoing progress in the field of GW,^5^ performing these calculations is not straightforward. The aforementioned scaling of  can still be an obstacle, and the calculations themselves can be challenging: they converge slowly with respect to the number of empty states included (which increases the importance of constructing transferable pseudopotentials that avoid ghost states^6^), and there is a strong interdependence of the results on different calculation parameters, which makes achieving convergence challenging at best. This hampers routine applications of GW (especially in a high-throughput context, where the calculations must be unsupervised).^7^ Finally, while in principle GW and many-body perturbation theory are systematically improvable—that is to say, by increasing the number of diagrams included in the calculations, the results should progressively converge to the correct answer (with GW outperforming GW_0_ in turn outperforming G_0_W_0_)—in practice this does not appear to hold.^8^

Alternatively, one could try and calculate the energies of electronic excitations with density-functional theory (DFT).^9,10^ DFT has proven to be a remarkably successful theory for predicting the ground-state properties of solids, surfaces, nanoparticles, and molecules.^11,12^ It is typically inexpensive, and these days such calculations are generally robust and can be treated as a “black box”. However, DFT is a theory of total energies, and while the Kohn–Sham auxiliary system is a powerful construct, the Kohn–Sham eigenvalues are not necessarily related to the energies of charged excitations (with the exception of the highest occupied molecular orbital, or HOMO, which is related to the exponential decay of the density^13^). Nevertheless, these eigenvalues can bear qualitative or even quantitative resemblance to experimental quasiparticle energies, and it is common practice to interpret them as such, motivated by the fact that the Kohn–Sham potential is the best local and static approximation to the electronic self-energy.^14^ Aside from this theoretical disconnect, problems also arise from the additional approximations inherent in exchange-correlation functionals. In the case of local and semilocal functionals, a key qualitative failure arises from the erroneous convex curvature in the total energy as a function of the total number of electrons in the system, which should instead be piecewise-linear.^15^ This curvature explains in part the disagreement between first ionization potentials as calculated via total energy differences compared to Kohn–Sham eigenvalues.

Many strategies have emerged that attempt to restore the piecewise linearity of the energy functional — the hope being that the resulting Kohn–Sham eigenvalues will yield accurate excitation energies. For example, DFT+*U* imposes a penalty functional to a localized subspace that restores linearity in the energy with respect to the occupation of this subspace.^16,17^ Similarly, hybrid functionals mix semilocal functionals with Hartree–Fock exchange (which happens to exhibit a concave curvature), which means that for a specific mixing fraction of the two functionals there will be an overall error cancellation.^18−21^ Recent state-of-the-art approaches that employ curvature corrections to yield reliable quasiparticle energies include screened, range-separated, and dielectric-dependent hybrid functionals with tuned mixing or range-separation parameters,^22−27^ as well as the Koopmans–Wannier method of Wang and co-workers^28^ and the localized orbital scaling correction (LOSC) of Yang and co-workers.^29−31^ Piecewise linearity is also central to ensemble density functional theory.^32,33^ Even DM21, the recent machine-learned exchange-correlation functional created by Google DeepMind, was constructed around the idea of restoring piecewise linearity.^34^

Starting in 2009, we have introduced and developed the concept of Koopmans functionals.^35−47^ By imposing a generalized piecewise linearity condition and relating quasiparticle energies to total energy differences, these functionals address the above issues, and as a consequence they yield spectroscopic properties (such as molecular ionization potentials, electron affinities, solid-state band structures, and band-edge alignments) with comparable accuracy to state-of-the-art GW approaches, but at greatly reduced computational cost while preserving a functional formulation. This has all been implemented in koopmans, an open-source package that allows nonexperts to perform their own Koopmans functional calculations, and which is built upon the popular Quantum ESPRESSO distribution. This paper provides an overview of the theory of Koopmans functionals (Section 2), describes the algorithms that enable their implementation in the koopmans package (Section 3), and demonstrates how these tools can be deployed to predict spectral properties using the examples of ozone, silicon, and zinc oxide (Section 4).

## 2 Koopmans Functionals

### 2.1 Fundamental Concepts

For a spectral theory, the orbital energies ε_*i*_ should match the total energy differences corresponding to electron removal *E*(*N*) – *E*_*i*_(*N* – 1) and addition *E*_*i*_(*N* + 1) – *E*(*N*). This is trivially true for the exact Green’s function, whose poles correspond directly to these total energy differences, but there is no such connection in Kohn–Sham DFT. The only exception to this is the HOMO, but even there the violation of piecewise linearity in density functional approximations leads to a mismatch between the HOMO eigenvalue with the corresponding total energy difference (i.e., the negative of the ionization potential).

Koopmans functionals restore this correspondence, by imposing the condition that the orbital energies  of orbitals φ_*i*_ should be independent of that orbital’s occupation *f*_*i*_:
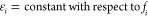
1It follows from Janak’s theorem that this is equivalent to a “generalized” piecewise linearity condition where the total energy of the system is piecewise linear with respect to the change of occupation of *any* orbital. This is a sufficient but not a necessary condition to fulfill the much more well-known piecewise linearity condition,^48^ which states that the total energy of the system is piecewise linear with respect to its *total* number of electrons. In passing, we mention that eq 1 is reminiscent of a photoemission experiment, where an electron is removed from a Dyson orbital.

Imposing this condition will require a beyond-DFT approach, and is not simply a matter of correcting density functional approximations within a DFT framework. We can see that this must be the case by considering the exact density functional, for which the Koopmans corrections must be nonvanishing. (This is because while the negative of the HOMO energy for the exact density functional matches the ionization potential, there is no such guarantee for the other eigenenergies.^13^)

The generalized piecewise linearity condition of eq 1 is imposed on a “base” functional (here, approximate or exact DFT) by removing, orbital-by-orbital, the nonlinear dependence of the energy *E* on the orbital occupation *f*_*i*_ and replacing it with a term that is linear in *f*_*i*_:
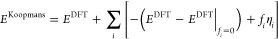
2where *E*^DFT^|_*f*_*i*_=*f*_ corresponds to the DFT energy of the (*N* – 1 + *f*)-electron system, with the occupancy of orbital *i* constrained to be *f*. The first term in the square brackets removes the dependence of the total energy on *f*, and the second term replaces it with a term explicitly linear in *f*. This construction is reminiscent of the SIC functional of ref (49)., but here the correction is generalized to the entire electronic manifold.

Here, one must choose a suitable slope η_*i*_ for this linear term; one option is to use the energy difference between fully occupied and empty orbitals
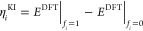
3giving rise to the Koopmans integer (KI) functional. Note that this formulation provides Koopmans functionals with meaningful eigenvalues, because they now correspond to total energy differences, which in the scope of DFT are formally meaningful and much more reliable than Kohn–Sham eigenvalues. It can be seen from eqs 2 and 3 that the KI functional gives, at integer occupations, the same total energy as the base functional, but has different derivatives and hence yields different spectral properties. (This will be discussed further in Section 2.4.1.)

Equations 2 and 3 are difficult to evaluate unless we only consider the explicit dependence of the DFT energy on the orbital occupancies, neglecting the implicit dependence of the orbitals φ_*i*_(**r**) on their own occupation *f*_*i*_, in which case
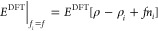
4where *n*_*i*_(**r**) = |φ_*i*_(**r**)|^2^ is the density of orbital *i* and ρ_*i*_(**r**) = *f*_*i*_|φ_*i*_(**r**)|^2^ = *f*_*i*_*n*_*i*_(**r**) is the occupancy-weighted density of orbital *i*. Orbital relaxation—or, equivalently, screening—is instead accounted for *post hoc* by scaling the unscreened correction by a scalar coefficient α_*i*_. Crucially, these coefficients can be calculated *ab initio* at the level of DFT via linear response or total energy differences.^43,44^ This brings us, finally, to the Koopmans energy functional:
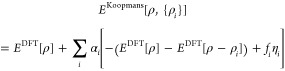
5

**Figure 1:**
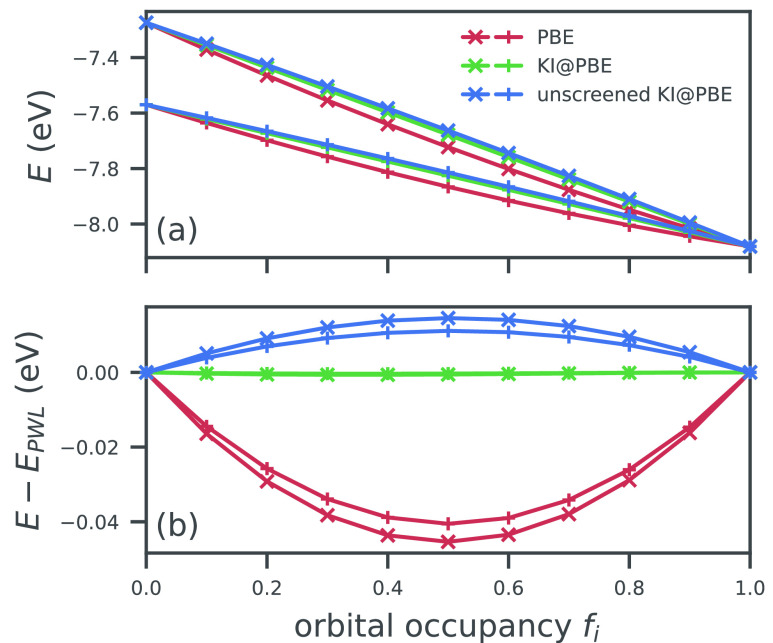
Total energy *E* of a CH_4_ molecule as a function of the occupancy of the 1a_1_ molecular orbital (crosses) and one of the 1t_2_ molecular orbitals (pluses). The absolute total energy is shown in panel (a) while the deviation from piecewise linearity is shown in panel (b). For both orbitals, PBE gives a total energy that is erroneously convex, while the KI correction successfully linearizes the total energy. Screening is key to this success; in its absence, the KI correction overcorrects the PBE base functional and yields a concave energy curve. Note that each orbital φ_*i*_ is obtained from the charge-neutral system (*f*_*i*_ = 1) and is frozen throughout (while all others are relaxed). If that orbital was not frozen, then as *f*_*i*_ → 0 the orbital would always morph into the LUMO of the *N* – 1-electron system and both sets of curves would be identical.

In Figure 1 we show the efficacy of this linearizing correction when applied to two orbitals in methane. The full derivation of eq 5 can be found in Supporting Information S1. This functional is actually very different from semilocal DFT functionals; this will be elaborated upon in the following sections.

### 2.2 Orbital-Density Dependence

The one important distinction that is worth making immediately is that Koopmans functionals are not density functionals, but *orbital-density-dependent* (ODD) functionals. This is because they are dependent on the individual orbital densities {ρ_*i*_} and not just the total electronic density ρ. A direct consequence of this is that Koopmans functionals — much like other ODD functionals such as the Perdew–Zunger self-interaction correction (PZSIC)—are no more invariant under unitary transformation of the occupied manifold, and their minimization requires extra care. The variation of *E*^Koopmans^ in eq 5 with respect to an arbitrary change of each orbital φ_*i*_ (density ρ_*i*_) leads to the Euler–Lagrange equations
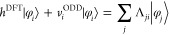
6where  is the Hamiltonian of the underlying DFT energy functional, *v*_*i*_^ODD^(**r**) is the orbital-density-dependent potential associated with the orbital φ_*i*_, and Λ_*ji*_ is the matrix of Lagrangian multipliers enforcing orthonormality constraints. Because of the ODD contribution, within the space spanned by the orbitals {φ_*i*_}) the energy is representation-dependent and a proper minimization of the functional requires its variation with respect to infinitesimal unitary transformations among the occupied orbitals to vanish,^40,50,51^ leading to the Pederson condition^50^
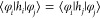
7The self-consistent solution of eqs 6 and 7 define the proper minimum of the Koopmans functionals, and the minimizing orbitals are known as the *variational* orbitals. The implementation of this minimization procedure will be discussed later in Section 3.1.

**Figure 2:**
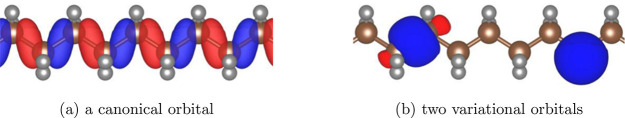
Example canonical and variational orbitals of polyethylene. Adapted from ref (43).

At the minimum, as a consequence of eq 7, the Λ matrix becomes Hermitian and can be diagonalized allowing us to define a set of *canonical* orbitals and energies. This mirrors the definition of canonical orbitals and energy in Hartree–Fock theory where, among all the equivalent sets of orbitals (those related by a unitary transformations) that minimize the functional, the canonical orbitals are recognized as those that also make the energy functional stationary when a fraction of electron is added to or removed from the system, thus qualifying these as electron addition/removal energies. This also applies to ODD functionals, as discussed in detail in ref (52) for the case of PZSIC. Moreover, canonical orbitals typically display the symmetry of the Hamiltonian operator (e.g., are Bloch states in periodic systems^45^ as shown in Figure 2a) and, in analogy to exact DFT, the energy of the highest occupied canonical orbitals has been numerically shown to determine the asymptotic decay of the ground-state charge density.^53^ For all these reasons, the canonical orbitals and the corresponding eigenvalues are usually interpreted as Dyson orbitals and quasiparticle energies. Nevertheless, it is important to stress that the reliability of canonical energies (and their correspondence with total energy differences) is not directly imposed by the Koopmans correction, but instead is inherited via the variational orbitals. That is to say: the Koopmans corrections are applied to the variational orbitals, and thus the Koopmans functional is linear with respect to the occupancy of variational orbitals. The canonical orbitals are composed of some linear combination of variational orbitals, and their energies (i.e., the quasiparticle energies) are subject to a weighted combination of corrective potentials arising from their constituent variational orbitals.

Given their central role in the theory, it is important to discuss the key features of variational orbitals. In contrast to canonical orbitals, variational orbitals are typically very localized in space (see Figure 2b). As was recognized long ago,^50^eq 7 is a localization condition that, once satisfied, leads to orbitals that resemble Boys orbitals in molecules or, equivalently, maximally localized Wannier functions in periodic systems.^54^ The localization of the variational orbitals is a common feature of ODD functionals and a key property for Koopmans functionals, in particular when it comes to dealing with periodic systems. By applying Koopmans corrections to a set of localized orbitals, the corrections are well-defined and nonvanishing for both small molecules, infinite bulk systems, and everything in between, preserving size-consistency.^43^ Contrast this to if we were to apply the corrections to the canonical orbitals, in which case they would become ill-defined in the bulk limit. In order to understand why this is the case, it is useful to return to the connection between the Koopmans construction and the ΔSCF approach. In a nutshell, the ultimate effect of the Koopmans correction is to revert the wrong eigenvalue from the underlying (approximate) density functional into a total energy difference (ΔSCF) between the neutral system and the system with plus or minus one electron evaluated using the same density functional. This means that the success of the approach relies on the quality of the ΔSCF value at the approximate DFT level. It is well-known that evaluating this total energy difference when removing an electron from a completely delocalized state reduces to the derivative of the total energy with respect to the particle number,^55−57^ which, for a local or semilocal density-functional approximation, is the negative of the KS-DFT eigenvalue. This means that for a standard density functional in the thermodynamic limit there is no difference between the ΔSCF and the KS eigenvalues and as a consequence the Koopmans corrections vanish. To overcome this issue, two routes are possible: either improving the base functional in such a way to have improved ΔSCF energies in the most general case, or retaining the simplicity of local and semilocal density-functionals and working in a localized representation of the orbitals.^28,58^ Indeed, the total energy differences of approximate density functionals also become accurate when computed on localized orbitals (e.g., typically, semilocal Δ*S*CF calculations accurately predict localized defect levels relative to the average electrostatic potential^59^). Thus, by applying the Koopmans corrections to the variational orbitals (and not the canonical orbitals), the Koopmans corrections are well-defined and nonvanishing also in the bulk limit, and yield accurate band structures compared to experiment. See ref (43) for more details.

Moving from a DFT framework to an ODDFT framework may appear like an unnecessary complication. This is not the case: ODDFTs are a very natural way to generalize a static functional theory like DFT to predict spectral information. Ultimately, the spectral properties of a many-body electronic system are exactly described by its nonlocal and dynamic self-energy. The exact Kohn–Sham potential is the best local and approximation to this self-energy.^14^ If we instead consider local but dynamic approximations, one enters into the domain of spectral functional theories, where the exact spectral functional predicts exactly the spectral density ρ(**r**, ω).^60^ ODDFTs can be interpreted as energy-discretized spectral functional theories,^39^ so as such an ODDFT framework is a sensible choice when attempting to predict spectral properties.

### 2.3 Accounting for Screening Effects

As discussed earlier in Section 2.1, we account for orbital relaxation *post hoc* via screening parameters {α_*i*_} and we can calculate these parameters *ab initio*. But how?

The crucial point is that we would like the total energy to be piecewise linear: that is, we would like orbital energies (specifically, the expectation value of the Hamiltonian on a given variational orbital) to match the corresponding total energy differences when adding/removing an electron from this orbital, without the frozen-orbital assumption that we made earlier. Specifically, we would like λ_*ii*_(α, *f*) = Δ*E*_*i*_^Koopmans^, where
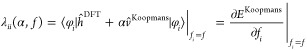
8is the expectation value of the Hamiltonian for a given variational orbital φ_*i*_, and
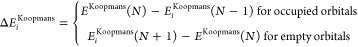
9where *E*_*i*_^Koopmans^(*N* ± 1) is the total energy of the system where we add/remove an electron from variational orbital *i* and allow the rest of the system to relax, with all the other orbitals remaining orthogonal to |φ_*i*_⟩.

We use this condition to determine the screening parameters *ab initio*. Specifically, given a starting guess {α_*i*_^0^} for the screening parameters, an improved guess for the screening parameters can be obtained via
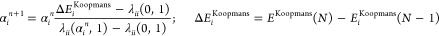
10for occupied orbitals and
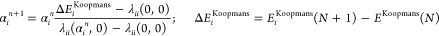
11for empty orbitals, where *E*_*i*_^Koopmans^(*N* ± 1) is the total energy of the *N* ± 1 electron system where we take the *N*-electron system, take this variational orbital *i* and fill/empty it, and then hold it frozen while the rest of the system is allowed to relax (while remaining orthogonal). These equations yield the screening parameters that satisfy λ_*ii*_(α, *f*) = Δ*E*_*i*_^Koopmans^ if we assume a linear dependence of λ_*ii*_ on α_*i*_ and approximate the total energy as a function of *f*_*i*_ to second order. By iterating to self-consistency we lift these approximations and guarantee that λ_*ii*_(α, *f*) = Δ*E*_*i*_^Koopmans^ is satisfied. Typically, only a few iterations are required in order to reach self-consistency, especially if one starts from a physically motiviated initial guess (such as the static limit of the inverse dielectric function ε^–1^ in the case of bulk systems). All of these ingredients for calculating α_*i*_^*n*+1^ are obtained from constrained Koopmans and DFT calculations. Specifically, a *N*-electron Koopmans calculation yields *E*^Koopmans^(*N*) and λ_*ii*_(α, *f*) (for both α = α_*i*_^*n*^ and 0, and *f* = 1 for filled orbitals and 0 for empty). Meanwhile, a constrained *N* ± 1-electron calculation yields *E*_*i*_^Koopmans^(*N* ± 1).

For a periodic system, this method for determining the screening parameters requires a supercell treatment. This is because the *N* ± 1-electron systems contain a charged defect (because we have filled/emptied a localized orbital) and a supercell is required in order to remove the spurious interactions between periodic images.^43,45^Section 3.3 will discuss an efficient linear-response reformulation of this problem that avoids a supercell treatment (and can also be used for molecules).

### 2.4 Koopmans Variants

As we saw previously in Section 2.1, there is some freedom in how one defines a Koopmans functional. Namely, one must choose values for η_*i*_, the gradient of the energy as a function of the occupancy of orbital *i*, for each value of *i* (modulo the corresponding screening term). In that section, we briefly introduced the Koopmans integer (KI) approach (eq 3), but that is just one of several different ways one can define these gradient terms, and it is possible to define several variants.

#### 2.4.1 KI

In the KI approach, η_*i*_ is chosen as the total energy difference of two adjacent electronic configurations with integer occupations as given by the base DFT functional:
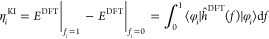
12where *ĥ*^DFT^(*f*) is the DFT Hamiltonian with the occupancy of orbital *i* constrained to *f*. In this case, the explicit expression for the unscreened KI Koopmans’ correction to orbital *i*, which we denote as Π_*i*_^KI^, becomes
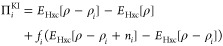
13where ρ_*i*_(**r**) = *f*_*i*_|φ_*i*_(**r**)|^2^ and *n*_*i*_(**r**) = |φ_*i*_(**r**)|^2^. *E*_Hxc_ denotes the Hartree and exchange-correlation energy corresponding to the underlying base functional.

It can be seen that at integer occupations the KI energy correction vanishes; that is, Π_*i*_^KI^ = 0. In other words, for integer occupations the KI functional preserves the potential energy surface of the base functional! But while the energy correction is vanishing, the potential is nonvanishing—for example, the KI potential correction to an occupied variational orbital is
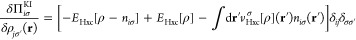
14(here the spin index σ has been decoupled from the orbital index). Unlike the energy correction in eq 13, this term is nonzero, which means that the KI correction will affect the spectral properties of the system while leaving the total energy unchanged.

#### 2.4.2 KIPZ

In the KIPZ approach the slope η_*i*_ is also chosen as the total energy difference of two adjacent electronic configurations with integer occupations, but this time using the Perdew–Zunger (PZ) one-electron-self-interaction corrected (SIC) functional applied to the approximate DFT base functional
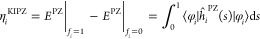
15In this instance, the explicit expression for the unscreened energy correction corresponding to orbital *i* (denoted Π_*i*_^KIPZ^) becomes
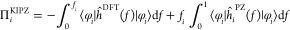
16where
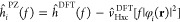
17is the PZ self-interaction correction applied to the *i*^th^ variational orbital with constrained occupation *f*, which removes the Hartree-plus-exchange-correlation potential for that orbital. The KIPZ correction can be rewritten as
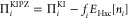
18which makes the physics of this correction clear: it is nothing less than the KI correction with the addition of a (screened) Perdew–Zunger self-interaction correction. This added correction removes one-electron self-interaction and makes the KIPZ functional exact for one-electron systems. In the many-electron case, it provides different (and typically improved) total energies and forces than the base functional,^41^ albeit with a screening coefficient for the Perdew–Zunger correction that is inherited from a spectral condition. More details are provided in Supporting Information S2.

#### 2.4.3 Comparing KI and KIPZ

The KIPZ correction is more computationally expensive than the KI approach, for the following reasons: we have already mentioned that the KI energy correction vanishes for integer orbital occupations. Furthermore, for occupied orbitals, the KI corrective potential is scalar (i.e., it does not have a spatial dependence) and therefore the total energy is invariant with respect to unitary rotations of the variational orbitals, provided we are at the minimum of the DFT energy. Consequently, once the occupied variational orbitals (and, by extension, the total density) are initialized they do not require further optimization. This also implies that the screening parameters of occupied variational orbitals converge instantly (in eq 10, Δ*E*_*i*_ and λ_*ii*_(0, 1) are independent of α_*i*_^*n*^ and λ_*ii*_(α_*i*_^*n*^, 1) is linear in α_*i*_^*n*^). Contrast this with KIPZ: the KIPZ energy does not match that of the base functional, nor is it invariant with respect to the unitary rotations of occupied orbitals. This means we must directly minimize the energy with respect to the shape of the variational orbitals, greatly increasing the computational cost of these calculations. Furthermore, the KIPZ ground-state density and variational orbitals are a function of the screening parameters, which means that the screening parameters must be calculated self-consistently, further increasing the computational cost.

Despite its additional computational cost, KIPZ has some desirable advantages over KI: for instance, it is one-electron-self-interaction-free. For this reason, we also have introduced the “perturbative KIPZ” (pKIPZ) method, where the KIPZ Hamiltonian is applied non-self-consistently to the KI density and variational orbitals, as a way of approximating the KIPZ result at reduced computational cost without significantly compromising the accuracy.^46^

It is important to note that the KI functional’s invariance with respect to unitary rotations of the occupied variational orbitals introduces an ambiguity in its definition: the variational orbitals are no longer well-defined. This ambiguity is resolved by formally defining the KI functional as the γ → 0 limit of the “KIγPZ” functional, which is the KIPZ functional with the PZ contribution to the correction scaled by a prefactor γ. This is discussed further in Supporting Information S3.1.1.

Finally, we note that the original formulations of Koopmans functionals also introduced the K and the KPZ functionals.^35,36,38^ These are similar to the KI and KIPZ functionals, except that the slope η_*i*_ is evaluated at half-occupation rather than as the total energy difference between integer occupations. These formulations provide almost identical results but more cumbersome than their integer counterparts.

#### 2.4.4 Total Energies and Forces with Different Koopmans Variants

The design of Koopmans functionals focuses on predicting spectral properties. However, it is worthwhile pausing to consider how accurately these functionals will predict structural properties (namely, total energies and forces). The KI functional, as we have already discussed, yields the same total energy—and by extension, the same forces—as its base functional. The KIPZ functional, on the other hand, gives total energies and forces that correspond to its base functional augmented with a screened PZ correction.

There are instances where these two approaches yield significantly different results. For example, in a study of the geometry of adenine, thymine, and uracil, the KIPZ@PBE functional predicted bond lengths with a relative mean absolute error compared to experiment of 0.65%, which was slightly better than PBE0 (0.76%) and PZ@PBE (0.83%), and was markedly better than PBE (1.63%)—and, by extension, KI@PBE.^42^ That same study showed that the KIPZ@PBE functional captured the tilt of the amino groups of nucleobases with respect to their aromatic rings, whereas PBE wrongly predicts a near-planar structure. However, the addition of a PZ correction does not necessarily improve structural properties across the board. Ref (38) compared structural properties for the reference G2-1 set of molecules, and found that KIPZ@PBE predicted bond angles less accurately (with a mean relative error of 2.2% for KIPZ@PBE compared to 1.4% for PBE) despite predicting bond lengths slightly better (1.5% for KIPZ@PBE compared to 2.3% for PBE).

We stress that these considerations regarding structural properties are somewhat orthogonal to the Koopmans functional formalism. One should not use the KI functional to calculate structural properties alone (because the ODD formalism comes at increased computational cost but provides no change in the structural properties). If desired, improved geometrical properties and accurate spectral properties can be simultaneously obtained by combining the KI correction with a more advanced base functional that predicts structural properties more reliably.

### 2.5 Important Caveats

Before concluding this section, there are a few further important points that must be made.

#### 2.5.1 Restriction to Systems with a Nonzero Band Gap

First, the Koopmans formulation is only well-defined for systems with a nonzero band gap. This is because the Koopmans correction (eq 8) is defined in terms of the diagonal elements of the occupation matrix. A band gap (however small) means that the occupancy matrix is block-diagonal, and can always be chosen to be the identity for the occupied manifold and zero for the unoccupied manifold. In the absence of a band gap, the occupancy matrix is not block-diagonal and a well-defined Koopmans functional would require some (currently unknown) corrections for the off-diagonal components. While it would be desirable to derive an off-diagonal correction and to lift this restriction, the current theory remains powerful—after all, it is in insulating and semiconducting systems where DFT exhibits one of its most striking failures in the underestimation of the band gap.

However, we note that we often rely on semilocal DFT as the base functional to define or initialize the variational orbitals. If the base functional also predicts a nonzero band gap, then the valence and conduction manifold can be disentangled,^61^ the occupancy matrix will be block-diagonal, and the Koopmans correction can immediately be applied. However, if the base functional wrongly predicts a metallic state, then the valence and conduction manifolds are not so easily disentangled. In these cases, one might be able to first employ other base functionals to open a gap (such as DFT+*U*) or deploy novel projectability disentanglement methods to separate the valence and conduction manifolds.^62^

The occupancies of variational orbitals *f*_*i*_ have been a central quantity in constructing the Koopmans formalism. This restriction to systems with a band gap means that these variational orbital occupancies will always be either 0 or 1, and consequently some terms in the formalism vanish (for example, the KI correction to the energy; eq 13) but others do not (for example, the KI correction to the potential; eq 14).

#### 2.5.2 Empty State Localization in the Bulk Limit

While minimizing the Koopmans energy functional for bulk systems leads to well-localized occupied orbitals, the same process does not lead to well-localized empty orbitals. This is because (a) low-lying conduction bands are often entangled with highly delocalized nearly free-electron bands, and (b) the Koopmans correction to empty states contains a leading Hartree term that incentivizes delocalization (see ref (38)). However, the Koopmans correction ought to be applied to localized orbitals, and vanishes in the limit of infinitely delocalized states (as discussed in Section 2.2). In light of this, we typically apply the Koopmans correction non-self-consistently on a maximally localized Wannier function representation of the empty manifold. This approach is heuristic but effective, as demonstrated by previous works.^43,45^

#### 2.5.3 Symmetries

Because a Koopmans potential *v*^Koopmans^[ρ, ρ_*i*_] is constructed via a variational orbital density, these potentials can break the translational symmetry of periodic systems. However, the variational orbitals crucially possess the translational properties of Wannier functions; that is, for each variational orbital φ_**R**_ there exists a periodic replica φ_**R+R**′_ such that
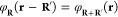
19where **R** and **R**′ can be any pair of Bravais lattice vectors. Thanks to this property, the collective potential∑_*i*_*v*^Koopmans^[ρ, ρ_*i*_]|φ_*i*_⟩⟨φ_*i*_| inherits the translational symmetry of the overall system and thus it remains possible to describe the system’s electronic structure with a band-structure picture. For more details, refer to ref (45).

More generally, the orbital-density dependence of Koopmans functionals might unphysically break the crystal point group symmetry. This is a common feature of nonrotationally invariant methods that are based on localized orbitals.^53,63^ Here, the symmetry of the localized representation plays an important role, especially in small systems and in the atomic limit. Possible solutions to this issue have been recently suggested,^64^ and this point is worthy of further investigation.

## 3 Algorithms and Implementation

The formulation of Koopmans functionals, as outlined in the previous section, is inherently more complex than a “standard” semilocal DFT calculation, and requires nonstandard algorithms and bespoke implementation within electronic-structure codes. This section describes these algorithms and describes how Koopmans functionals have been implemented in Quantum ESPRESSO and the open-source package koopmans.

### 3.1 Orbital Optimization

In order to work with Koopmans functionals, we must be able to minimize an orbital-density-dependent functional. In other words, we must optimize a set of orbital densities {ρ_*i*_} such that the Koopmans energy functional (eq 5) is minimized. This orbital optimization is performed separately for the occupied and then the empty manifold using an optimization algorithm similar to that employed in the ensemble DFT approach:^65^ the orbital densities are parametrized via a set of wave functions ϕ_*i*_ and a unitary rotation matrix *U*, such that ρ_*i*_ = |(*U*ϕ)_*i*_|^2^, and then the energy is then minimized via the nested loop:
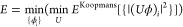
20where in the inner loop the unitary rotation matrix *U* is optimized (which leaves the total density unchanged), and in the outer loop the wave functions are optimized. Both steps are performed using the conjugate-gradient algorithm. The optimization is performed separately for the occupied and empty manifolds to ensure that the occupation matrix remains block-diagonal (as discussed in Section 2.5.1).

One important ingredient in ODD energy minimization is the use of complex orbitals. Because the ODD energy is not invariant with respect to unitary rotations of the variational orbitals, it can no longer be assumed (as in the case for DFT) that the variational orbitals are real, and thus the aforementioned wave functions ϕ_*i*_ must be complex in order to find the true minimum of the ODD functional.^38,63,66−68^

In addition to the generic orbital minimization procedure, we must also perform constrained minimization calculations (as required by the finite-difference method for calculating screening parameters; Section 2.3). Here, the total ODD energy is minimized while removing/adding one electron to a particular variational orbital. (This gives us *E*_*i*_(*N* ± 1) from eq 9). This orbital must be frozen during the minimization, otherwise it would morph into the valence band maximum/conduction band minimum, and one must also impose the standard orthogonality condition with all other orbitals belonging to the same spin channel. Image correction methods such as Martina-Tuckerman or Gygi-Baldereschi^69,70^ must be used to avoid spurious interaction between charged periodic images. For periodic systems this also means that these calculations must be performed in a supercell. These charged defect calculations also require special care in low-dimensional materials.^71^ Further details regarding the orbital minimization procedure are presented in ref (40).

### 3.2 The kcp.x Code

These orbital minimization algorithms are implemented in the code kcp.x. In other words, kcp.x can be used to obtain the ground-state energy and the minimizing set of variational orbitals of an arbitrary system for a given orbital-density-dependent functional (PZ, KI, or KIPZ).

kcp.x can be used to calculate screening parameters via the finite-difference approach, and is applicable to both periodic and aperiodic systems. By design, it does not use **k**-point sampling for periodic systems, because the finite-difference approach mandates the use of a supercell (as discussed above), rendering **k**-point sampling of the Brillouin zone superfluous. Instead, the dimensions of the supercell can be used to effectively sample **k**-space for bulk systems, and the band structure for the equivalent primitive cell can be reconstructed at the end of the calculation using an unfolding procedure.^45^ Despite the absence of **k**-space sampling (which is embarrassingly parallel), the kcp.x code still uses MPI parallelism: it is parallelized over the plane wave basis. This allows for the distribution of linear algebra operations and Fourier transforms across processors.

Because Koopmans functionals are a correction applied on top of a local or semilocal functional, and these functionals are computationally inexpensive compared to their ODD counterparts, before commencing orbital minimization with kcp.x it is efficient to initialize the variational orbitals as Kohn–Sham orbitals or maximally localized Wannier functions.^54^ To support the use of Wannier functions for periodic systems, we have implemented an interface that takes set of **k**-indexed Wannier functions from a Wannier90 calculation and maps it to an enlarged set of Γ-only Wannier functions defined on the corresponding supercell. Given that kcp.x implements the full minimization of the ODD functional, in principle one could use the output of kcp.x to perform geometry optimizations, calculate phonons via the frozen-phonon method, calculate electron–phonon coupling, model excitons, and so on.

For historical reasons, kcp.x is implemented on top of cp.x, the code within Quantum ESPRESSO usually responsible for performing Car–Parrinello molecular dynamics (hence the name “kcp.x”), which already contained algorithms similar to the direct functional minimization required by Koopmans functionals. It is important to note that kcp.x is not meant to perform molecular dynamics like cp.x. The implementation is built on top of version 4.1 of Quantum ESPRESSO. The modifications made to implement Koopmans functionals are (a) extensive and (b) of no relevance to the standard functioning of the cp.x code, so these modifications have not yet been incorporated within the official Quantum ESPRESSO repository, nor was the private version of the code kept aligned with subsequent Quantum ESPRESSO releases. Fast-forward to today, and kcp.x has effectively become a standalone code.

### 3.3 Screening Parameters via Linear Response Calculations in Reciprocal Space

While the finite-difference approach of kcp.x can provide us with all of the ingredients to calculate the screening parameters, it is somewhat cumbersome, since one must perform several constrained DFT and Koopmans calculations, and for periodic systems these must be performed in a supercell. An alternative to this approach is to compute the screening coefficients via density-functional perturbation theory (DFPT).^72^

In this approach, one first approximates the energy as a quadratic function of the occupation number (which is typically a very good approximation), and the expression for the screening coefficients reduces to
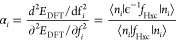
21where  () represents variations that do (do not) account for orbital relaxation, ϵ(**r**, **r**′) is the microscopic dielectric function of the material, *f*_Hxc_(**r**, **r**′) = δ^2^*E*_*Hxc*_/*δρ*(**r**)*δρ*(**r**′) is the Hartree-plus-exchange-and-correlation kernel, and *n*_*i*_(**r**) = |φ_*i*_(**r**)|^2^ is the orbital density at integer occupation.^44^ This can be evaluated by considering the density response Δ^*i*^*n*(**r**) induced in the system by the perturbing potential *v*_pert_^*i*^(**r**) = ∫*d***r**′*f*_Hxc_(**r**, **r**′)*n*_*i*_(**r**′). This perturbation is the Hartree-plus-exchange-and-correlation potential generated when adding/removing an infinitesimal fraction of an electron to/from orbital *i*. One determines Δ^*i*^*n* self-consistently via DFPT,^73^ and then the screening parameters are given by
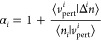
22Evaluating the screening coefficients within this linear-response approach only requires quantities available from a *N*-electron calculation, which means that in the case of periodic solids there is no need for a supercell. Instead, we can reduce the cost of these calculations by taking advantage of the translational symmetry of the system^45^ and recasting the supercell problem in a basis of Wannier functions. These Wannier functions take the form *w*^**R** i^(**r**), where the orbital label explicitly denotes the lattice vector **R** of the home cell inside the supercell. In this basis, the DFPT expression for the screening coefficients (eq 22) can be decomposed into a set of independent problems (monochromatic perturbations), one for each **q** point sampling the Brillouin zone of the primitive cell.^73^ The now **q**-dependent charge density variation Δ*n*_**q**_^**0***i*^(**r**) induced by the perturbing potential *v*_pert,**q**_^**0***i*^ is obtained self-consistently via DFPT (eqs 15–17 of ref (73)), and then the screening coefficients are obtained by summing over **q**:
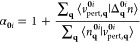
23The KI Hamiltonian at a particular **k** point is then given to second order by
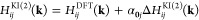
24where the second-order KI contribution to the Hamiltonian is
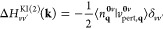
25for valence bands and
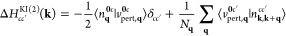
26for conduction bands, where *n*_**k**,**k**+**q**_^*cc*′^(**r**) = (*w*_**k**_^*c*^(**r**))*w_**k**+**q**_^*c*′^(**r**); *w*_**k**_^*c*^(**r**) is the periodic part of the electronic state in the Wannier gauge. As expected, the KI contribution to the valence bands is **k**-independent. The total Hamiltonian is then diagonalized in order to obtain the canonical eigenstates and energies. Given the fact that the Hamiltonian is written in a basis of Wannier functions, it is also possible to employ standard interpolation techniques to obtain the KI eigenvalues at any arbitrary **k**-point.^54^

However, the DFPT approach does come with some limitations. The principal limitation is that the energy is approximated to second order in the perturbing potential. In most cases this is very accurate, correctly capturing the quadratic Hartree contribution and only missing the nonquadratic, higher-order exchange-correlation contributions.

### 3.4 The kcw.x Code

The calculation of screening parameters via DFPT and the subsequent construction of the Koopmans Hamiltonian and band structure, as described above, has been implemented in the code kcw.x. Because all of these calculations are performed in a basis of Wannier functions, this code obtains Wannier functions via an interface with Wannier90. (The “w” in kcw stands for “Wannier”.) Because all of these equations are formulated in terms of a primitive cell with **k**-point sampling, kcw.x uses MPI to parallelize over **k**-points. It also parallelizes over plane-wave orbitals (as already introduced in the context of kcp.x).

While much of the above applies to periodic systems, kcw.x can still be used to perform calculations on aperiodic systems. The Wannier function basis still remains valid, but we no longer have multiple **k**-points.

kcw.x is part of the official Quantum ESPRESSO distribution (from version 7.1 onward).

### 3.5 Comparing kcp.x and kcw.x

kcp.x and kcw.x implement different Koopmans strategies and, as such, they have different use-cases, largely defined by their computational scaling. The two codes scale differently largely due to the fact that kcw.x operates in a primitive cell while kcp.x operates in a supercell. Calculating one screening parameter using kcp.x requires multiple SCF calculations, each of which takes a computational time *T*^SC^ that roughly scales as , where *N*_orb_^SC^ is the number of orbitals in the supercell. Meanwhile, calculating one screening parameter using the kcw.x DFPT approach scales as *T*^PC^ ∝ *N*_**q**_*N*_**k**_*N*_orb_^PC^3^^. This is the typical computational time for the SCF cycle *N*_**k**_*N*_orb_^PC^3^^ times the number of independent monochromatic perturbations *N*_**q**_. Using the relation *N*_orb_^SC^ = *N*_**k**_*N*_orb_^PC^ and the fact that *N*_**q**_ ≲*N*_**k**_, the ratio between the supercell and primitive computational times is roughly proportional to *N*_**q**_. Thus, as the supercell size (or equivalently the number of **q**-points in the primitive cell) increases, the kcw.x DFPT approach becomes more and more computationally efficient.^73^ For aperiodic systems, *N*_**q**_ = 1 and the two approaches scale similarly, but with different prefactors.

Note that these scaling relations pertain to the calculation of a single screening parameter, whereas a full Koopmans workflow requires the calculation of one screening parameter per unique variational orbital in the system. Here, the word “unique” is very important; orbitals that are related by symmetry will share the same screening parameter and therefore the screening does not need to be recalculated for each orbital. This means that in the worst-case scenario, where none of the variational orbitals are related by symmetry, the overall scaling of the workflow has an additional *N*_orb_ prefactor, but for many systems (and for periodic systems in particular) the number of unique variational orbitals in the system can be many times smaller than the total number of orbitals. Furthermore, (a) the calculation of screening parameters for separate orbitals is embarrassingly parallelizable, and (b) it is possible to predict the screening parameters via machine learning, avoiding the need to repetitively calculate screening parameters altogether.^74^

The superior scaling of kcw.x comes at a cost, as it makes two approximations that kcp.x does not: the DFPT approach expands the total energy only to second order when computing screening parameters (see Section 3.3), and it does not optimize the variational orbitals. These are instead defined via Wannier functions, which often closely resemble the minimizing orbitals of the Koopmans energy functional. This also means that kcw.x only implements the KI functional. Without orbital minimization one cannot perform KIPZ calculations, and pKIPZ would require the PZ kernel (i.e., the second derivative of the PZ energy with respect to the density), and this is not implemented in common electronic-structure codes.

### 3.6 Workflow Management

Running a Koopmans calculation with either kcp.x or kcw.x requires a few additional steps compared to a standard semilocal DFT calculation. In this section, we will focus on the workflows that one needs to perform in order to complete a Koopmans functional calculation, and how these are publicly disseminated in open-source form.

Typically, these workflows can be divided into three steps:

1. an initialization step, where the variational orbitals are initialized
2. the calculation of screening parameters
3. a final calculation using the final screening parameters

Depending on the method used for calculating screening parameters (that is, either finite differences with kcp.x or DFPT with kcw.x), the resulting workflows look very different. Differences also emerge between calculations on molecules and solids. For the latter (and for large molecular systems), we have already seen that maximally localized Wannier functions are typically used as the variational orbitals (for KI) or as a starting guess for the variational orbitals (for KIPZ). This necessitates an additional Wannierization procedure^54^ and an interface between Wannier90 and kcp.x/kcw.x. Meanwhile, for calculating the screening parameters via finite differences, we must perform a combination of different constrained orbital minimizations. In all cases, the workflows typically comprise of several if not dozens of calculations, often involving different electronic structure codes that must handshake with one another. This can greatly benefit from automation.

### 3.7 The koopmans Package

These workflows are all implemented within the koopmans package. Users exclusively interact with koopmans, rather than the electronic structure codes directly (which can include, in addition to kcp.x and kcw.x, pre-existing codes such as pw.x, pw2wannier90.x, and wannier90.x(75−77)).

Typically, a user provides koopmans with a single input JSON file (some examples are provided in Supporting Information S4). Based on the settings provided in this input file, koopmans proceeds through the requested workflow. Whenever an electronic structure calculation needs to be performed, it generates the corresponding input file, calls the relevant code, waits for it to complete, and then parses the output file. Between successive calculations, it computes intermediate variables, moves and modifies files, etc. In other words, the workflow runner takes care of the banal aspects of performing a Koopmans calculation, allowing users to concern themselves with scientific matters (e.g., “what functional do I want to use?”) rather than getting bogged down in practical details (e.g., “are the Wannier function files in the correct format for the next calculation to be able to read?”)

The koopmans package is shipped with versions of Quantum ESPRESSO that contain kcp.x and kcw.x, meaning that it contains everything that is required to perform Koopmans functional calculations from start to finish.

Further details on the koopmans package can be found in Supporting Information S5. A step-by-step explanation of the workflows themselves can be found in Supporting Information S3.

## 4 Example Calculations

This Koopmans functional formalism has already proven to be very powerful. In ref (46), Koopmans functionals were found to predict the ionization potentials of a set of 100 small molecules with comparable/superior accuracy to state-of-the-art GW approaches. Importantly, Koopmans functionals do not only correct the ionization potential (i.e., the charged excitation where the most weakly bound electron is removed) but *any* single-particle charged excitation. This was shown for a large set of molecules relevant for photovoltaic applications,^41^ with Koopmans functionals yielding ultraviolet photoemission spectra that agree quantitatively with experiment. One can see similar accuracy in the prediction of band gaps and band structures of periodic systems;^43,45,73^ in a study of prototypical semiconductors and insulators, Koopmans functionals were found to yield band gaps with a mean absolute error of 0.22 eV, compared to 0.18 eV when using self-consistent GW with vertex corrections.^43^ Importantly, alignment between the valence band edge and the vacuum level was also very good: across six semiconductors the mean absolute error was 0.19 eV, compared to 0.39 eV for G_0_W_0_ and 0.49 eV for self-consistent GW with vertex corrections. Finally, Koopmans functionals can accurately describe the spectral properties of liquids, with the KIPZ functional predicting the electronic density of states of liquid water with comparable accuracy to self-consistent GW with vertex corrections.^78^

However, all of these calculations were performed by individuals with expert knowledge of Koopmans functionals and with specific expertise on the codes that implement them. This final section demonstrates the capabilities of the koopmans package by way of several examples. All of the following calculations are possible using a very minimalist input file (see Supporting Information S4). Note that the following calculations use slightly underconverged parameters (specifically, the energy cutoff, cell size, and/or the size of the *k*-point grid). Our focus here is to provide example calculations that can be reproduced easily by readers, rather than providing high-quality reference results.

### 4.1 The Ionization Potential and Electron Affinity of Ozone

First, we present the calculation of the ionization potential and electron affinity of ozone using koopmans.

This calculation is run with the simple command koopmans ozone.json; the input and output files for which can be found in Supporting Information S4.1. In short, this command prompts the full sequence of Quantum ESPRESSO calculations necessary to initialize the density and variational orbitals, calculate the screening parameters, and run a final KI calculation. The Quantum ESPRESSO input and output files for these calculations are all stored in various subdirectories of the current working directory. In principle one can then simply parse the quantities of interest from the output files (but there are easier ways, as explained in Supporting Information S5.2). Refer to Supporting Information S3.1 for a detailed step-by-step description of this workflow.

The ionization potential (IP) and electron affinity (EA) of ozone, as given by this calculation, are listed in Table 1, showing the excellent performance of the KI functional compared to state-of-the-art methods.

**Table 1:** Vertical Ionization Potential (IP) and Electron Affinity (EA) of Ozone, As Calculated Using Functional, Perturbative, and Quantum Chemistry Methods, as well as Experiment

	IP	EA	
PBE	7.95	6.17	This work
G_0_W_0_^a^	11.80 ± 0.25	2.34 ± 0.25	Ref (79)
scGW_0_@PBE	12.57		Ref (80)
scGW_0_@HF	13.16		Ref (80)
scGW	12.54		Ref (80)
qsGW	13.21		Ref (80)
CCSD(T)	12.55		Ref (81)
KI@[PBE,KS]^b^	12.52	1.82	This work
	12.91		Ref (46)
experiment	12.73	2.10	Refs (82−85)

a The uncertainties in the G_0_W_0_ correspond to the standard deviation of values reported in ref (79), which presents calculations using a range of codes and basis sets.

b The KI correction on top of the PBE base functional, with Kohn–Sham orbitals defining the variational orbitals, showing excellent performance compared to state-of-the-art methods.

### 4.2 The Band Structure of Silicon

koopmans can also perform calculations on bulk systems. Here one typically performs a Wannierization procedure in order to generate maximally localized Wannier functions to use as variational orbitals. Running this calculation gives rise to a similar output to the previous case, with the notable exception that the initialization procedure now involves Wannierization (see Supporting Information S4.2).

**Figure 3:**
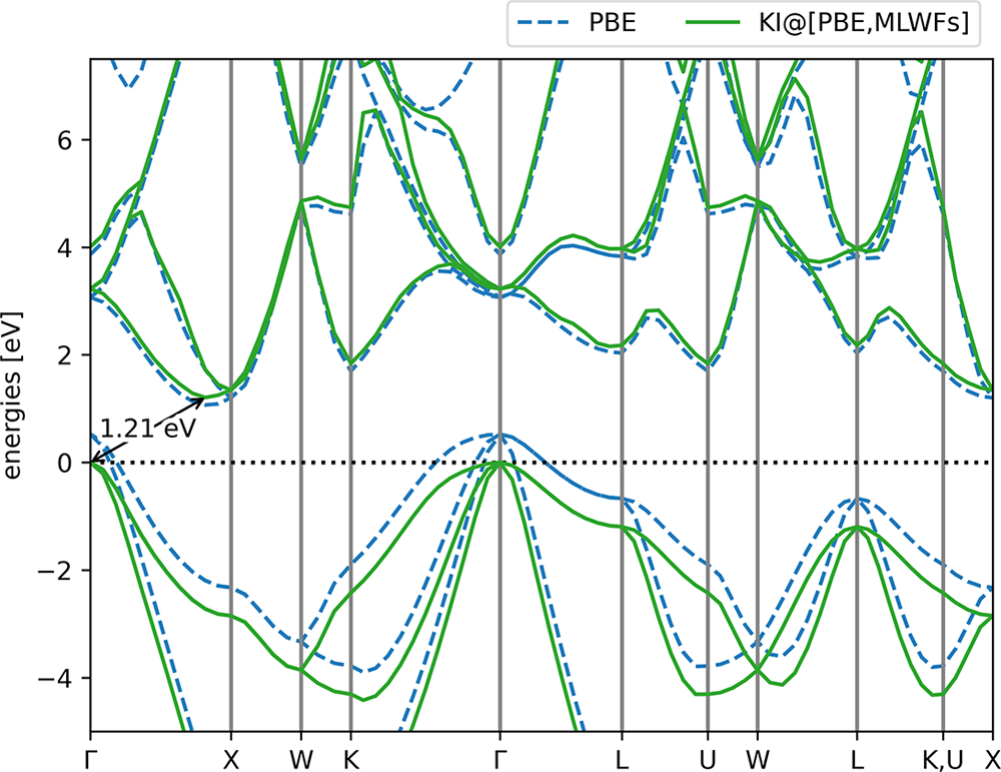
Band structure of bulk silicon, calculated using the KI functional with the PBE base functional, MLWFs as variational orbitals, and screening parameters calculated via finite differences. The PBE band structure is also plotted for comparison.

The band structure that one obtains from this calculation is shown in Figure 3, the band gap is displayed in Table 2, alongside energy differences between particular symmetry points in the band structure.

**Table 2:** Band Gap *E*_*g*_ and Energy Differences between Symmetry Points in the Band Structure of Bulk Silicon (in eV), Calculated with Various Functional and Perturbative Approaches^a^

				KI@[PBE,MLWFs]			
	PBE^b^	G_0_W_0_^c^	scGW̃^d^	This work	Ref (45)	KIPZ@PBE^e^	Exp^f^
*E*_*g*_	0.49	1.06	1.14	1.16	1.12	1.15	1.17
Γ_1*v*_ → Γ_25′*v*_	11.97	12.04		11.97	11.96	12.09	12.5 ± 0.6
*X*_1*v*_ → Γ_25′*v*_	7.82			7.82			7.75
*X*_4*v*_ → Γ_25′*v*_	2.85	2.99		2.85	2.84	2.86	2.90
*L*_2′*v*_ → Γ_25′*v*_	9.63	9.79		9.63	9.63	9.74	9.3 ± 0.4
*L*_1*v*_ → Γ_25′*v*_	6.98	7.18		6.98	6.96	7.04	6.8 ± 0.2
*L*_3′*v*_ → Γ_25′*v*_	1.19	1.27		1.19			1.2 ± 0.2
Γ_25′*v*_ → Γ_15*c*_	2.48	3.29		3.17	3.18	3.20	3.35 ± 0.01
Γ_25′*v*_ → Γ_2′*c*_	3.28	4.02		3.95	3.94	3.95	4.15 ± 0.05
Γ_25′*v*_ → *X*_1*c*_	0.62	1.38		1.28	1.30	1.31	1.13
Γ_25′*v*_ → *L*_1*c*_	1.45	2.21		2.12	2.12	2.13	2.04 ± 0.06
Γ_25′*v*_ → *L*_3*c*_	3.24	4.18		3.91	3.93	3.94	3.9 ± 0.1
MSE	0.35	0.02		0.01	0.00	0.03	
MAE	0.44	0.21		0.14	0.16	0.17	

a These are compared against experimental values via the mean signed and mean absolute errors (MSE and MAE respectively). The calculated values for the band gap and the valence-to-conduction transitions have been shifted by −0.06 eV to account for zero-point renormalization.^90^

b This work.

c Ref (86) for *E*_g_ and ref (87) for the transitions.

d Ref (88).

e Ref (45).

f Ref (89).

The experimental band gap is reproduced with accuracy comparable to self-consistent GW with vertex corrections, and the energy differences between symmetry points are reproduced with comparable accuracy to G_0_W_0_ (the only perturbative method for which these data were available). Note that the PBE and KI valence-to-valence energy differences match. This occurs because the occupied manifold is comprised of four identical Wannier functions, and thus the KI correction to these bands amounts to a rigid shift. Contrast this with the valence-to-conduction energy differences, which are markedly better for the KI functional.

### 4.3 The Band Structure of Zinc Oxide

**Figure 4:**
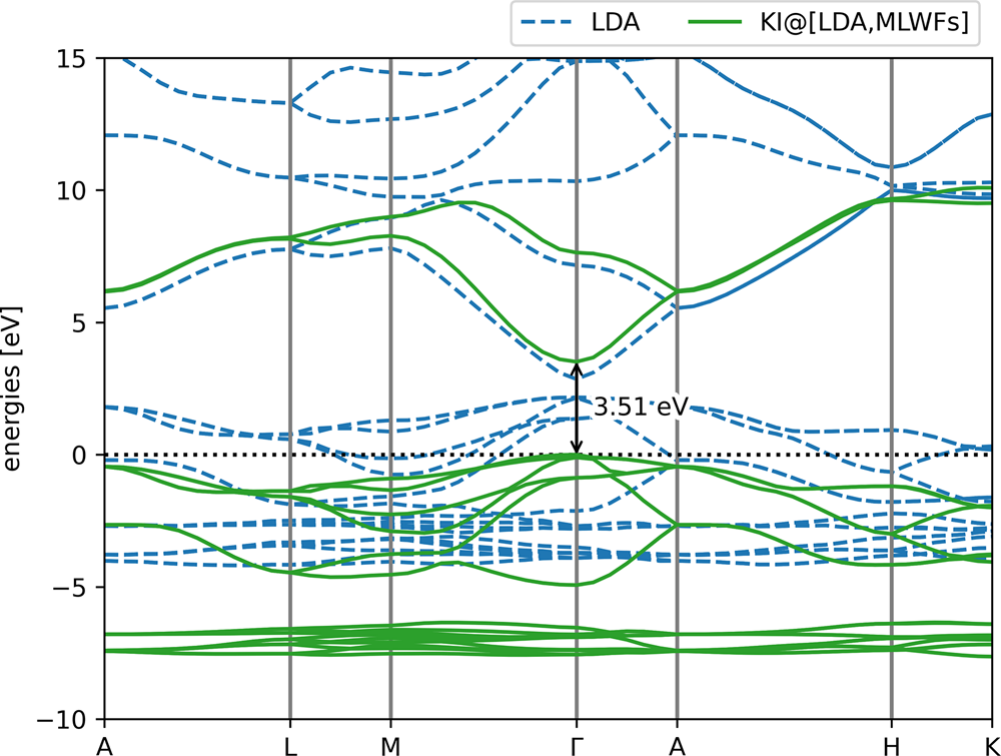
Band structure of zinc oxide, calculated using the KI functional with the LDA base functional, MLWFs as variational orbitals, and screening parameters calculated via DFPT. The LDA band structure is also plotted for comparison.

The previous example used the finite-difference approach for calculating the screening parameters. In this final example, we will instead use the DFPT approach to calculate the band structure of zinc oxide. Refer to Supporting Information S3.2 for a step-by-step description of what this entails. The calculated band structure is shown in Figure 4 and the band gaps are listed in Table 3. The corresponding input and output files are provided in Supporting Information S4.3. In this instance, the band gap is predicted with better accuracy than state-of-the-art self-consistent GW with vertex corrections. This is also true of the average *d*-band energy, although these bands remain slightly too high in energy relative to experiment. Finally, the bandwidth of the oxygen 2*p* bands (the six highest-energy occupied bands) is much improved going from LDA to KI. Note that this is a major departure from the earlier calculations on silicon, where the KI correction to the occupied bands amounts to a rigid shift and thus such a bandwidth would not change. In this instance, these bands comprise of variational orbitals of multiple different characters, each of which is subject to its own potential shift, and thus the overall band shape can (and does) change.

**Table 3:** Band Gap *E*_*g*_, Average *d*-Band Energy ⟨ε_d_⟩, and Bandwidth Δ of Bulk Zinc Oxide (all in eV), As Given by Various Functional and Perturbative Approaches, and Compared to Experiment^a^

	LDA^b^					KI@[LDA,MLWFs]^b^		
	This work	Ref (73)	G_0_W_0_^c^	GW_0_^d^	scGW̃^d^	This work	Ref (73)	Exp
*E*_g_	0.53	0.63	1.96	2.84	3.04	3.35	3.52	3.44^e^
⟨ε_d_⟩	–5.42	–5.14	–6.1	–6.4	–6.7	–7.06	–6.93	–7.5 to −8.81^f^
Δ	4.43	4.15				4.93	4.99	5.3^g^

a All of the computational values for the band gap have been shifted by −0.16 eV to account for zero-point renormalization.^90,94^

b In contrast to the earlier calculation on bulk silicon, here we used the LDA base functional to align with ref (73). That work used finer parameters than this work (most notably, a finer *k*-point grid throughout).

c Ref (86).

d Ref (88).

e Ref (91).

f Ref (92).

g Ref (93).

## 5 Conclusions

Koopmans functionals are a powerful computational tool for predicting the spectral properties of atoms, molecules, liquids, and crystalline and amorphous solids from first-principles with a functional approach. This has already been demonstrated in their ability to calculate the ionization potentials and electron affinities of small molecules,^41,46^ the photoemission spectra of large molecules,^41,42^ the electronic structure of liquid water,^78^ and the band structures and ionization potentials of prototypical semiconductors and insulators,^43,45,73^ all at a level of accuracy comparable to state-of-the-art many-body perturbation methods.

The newly released koopmans package now makes it possible, for the first time, for nonexperts to use these functionals in their own research. Experts will also benefit from their calculations becoming much more robust and reproducible. For more information, we refer the reader to the website koopmans-functionals.org.

The koopmans package will continue to be maintained and developed. In particular, Koopmans calculations on periodic systems require the user to perform a Wannierization of the electronic states, and correctly configuring this calculation can be onerous. In the near future we will add support for automated Wannierization.^61,62^

The second focus of ongoing development will be parallelization. Large swathes of the Koopmans workflow (for example, the calculation of screening parameters) are embarrassingly parallel. For example, one could calculate a revised value of the screening parameter for orbital *i* entirely independently of the calculation of the screening parameter for orbital *j*. (This is true for both the finite difference and DFPT schemes.) However, koopmans performs each calculation in the workflow serially, i.e., multiple calculations are not run simultaneously. (N.B. We are not saying that individual calculations must be run on a single core; all the codes support MPI parallelization.) Integration of the workflows within a workflow engine such as AiiDA would allow us to massively reduce the workflows’ walltimes.^95^ Integration within AiiDA would come with the added benefits of provenance tracking and error detection/recovery. Combined with the automated Wannierization and efficient parallelism, high-throughput studies with Koopmans functionals are just around the corner.

## Data Availability

All of the input and output files related to this paper can also be found on Materials Cloud at 10.24435/materialscloud:9w-sp.

## Supporting Information Available

A derivation of the functional form of Koopmans functionals, further details on the KIPZ functional, a detailed description of the Koopmans workflows, example input and output koopmans files, and additional details regarding the koopmans package (PDF)
