# Supplementary material for: koopmans: An Open-Source Package for Accurately and Efficiently Predicting Spectral Properties with Koopmans Functionals
Source: J Chem Theory Comput. 2023 Aug 23;19(20):7097–111. doi: 10.1021/acs.jctc.3c00652 (PMC10601481; doi:10.1021/acs.jctc.3c00652)
Supplement: Supplementary file 1 — ct3c00652_si_001.pdf [file ct3c00652_si_001.pdf]

Supporting Information:

koopmans: an open-source package for accurately and efficiently predicting spectral properties with Koopmans functionals

Edward B. Linscott,[*,†,△] Nicola Colonna,[‡,¶,△] Riccardo De Gennaro,[†] Ngoc Linh Nguyen,[§,‖] Giovanni Borghi,[†,∇] Andrea Ferretti,[⊥] Ismaila Dabo,[#] and Nicola Marzari[*,†,¶,@]

†*Theory and Simulation of Materials (THEOS), École Polytechnique Fédérale de Lausanne, 1015 Lausanne, Switzerland*

‡*Laboratory for Neutron Scattering and Imaging, Paul Scherrer Institut, 5232 Villigen, Switzerland*

¶*National Centre for Computational Design and Discovery of Novel Materials (MARVEL), École Polytechnique Fédérale de Lausanne, 1015 Lausanne, Switzerland*

§*Faculty of Materials Science and Engineering, Phenikaa University, Hanoi 12116, Vietnam*

‖*Phenikaa Research and Technology Institute (PRATI), A&A Green Phoenix Group JSC, No. 167 Hoang Ngan, Trung Hoa, Cau Giay, Hanoi 11313, Vietnam*

⊥*Centro S3, CNR–Istituto Nanoscienze, 41125 Modena, Italy*

#*Department of Materials Science and Engineering, Materials Research Institute, and Institutes of Energy and the Environment, The Pennsylvania State University, University Park, Pennsylvania 16802, USA*

@*Laboratory for Materials Simulations, Paul Scherrer Institut, 5232 Villigen, Switzerland*

△*Contributed equally to this work*

∇*Now at Liceo Manfredo Fanti, 41012 Carpi, Italy*

E-mail: edward.linscott@epfl.ch; nicola.marzari@epfl.ch

S1 Derivation of the functional form of Koopmans functionals

The brief derivation of the functional form of Koopmans functionals is as follows: let us assume a functional of the form

$$E^{\text{Koopmans}} = E^{\text{DFT}} + \sum_i \Pi_i \tag{S1}$$

If we take the derivative with respect to the occupancy of the jth variational orbital then we have

$$\eta_j = \left.\frac{dE^{DFT}}{df_j}\right|_{f_j=f} + \left.\frac{d\Pi_j}{df_j}\right|_{f_j=f} = \langle\varphi_j|\hat{h}^{\text{DFT}}(f)|\varphi_j\rangle + \left.\frac{d\Pi_j}{df_j}\right|_{f_j=f} \tag{S2}$$

where we assumed that the cross-term derivatives $d\Pi_i/df_j$ vanish, and because E^{Koopmans} ought to be linear in f_j, we replaced its derivative with some yet-to-be deterimined constant η_j. For the second equality we invoked Janak's theorem, and f is some number between 0 and 1.

Assuming that the energy correction Π_j is zero at integer occupancies, is independent of f_i for $i \neq j$, and neglecting for the moment any orbital relaxation as the orbital occupancies change, it follows that

$$\Pi^u_j = -\int_0^{f_j} \langle\varphi_j|\hat{h}^{\text{DFT}}(f)|\varphi_j\rangle df + f_j\eta_j = -\left(E^{\text{DFT}}[\rho] - E^{\text{DFT}}[\rho - \rho_i]\right) + f_j\eta_j \tag{S3}$$

where the u superscript denotes the fact that we neglected orbital relaxation, and thus this term is "unscreened". To account for this screening we must introduce some screening parameters $\{\alpha_i\}$ such that $\Pi_j = \alpha_j\Pi^u_j$. Having done this, we arrive at eq. 5 of the main text, the final result.

S2 KIPZ details

In previous works, KIPZ has been presented in slightly different ways. In eq. 27 of Ref. S1, KIPZ was introduced as

$$\Pi_i^{\mathrm{KIPZ}} = -\int_0^{f_i} \langle\varphi_i|\hat{H}_i^{\mathrm{PZ}}(s)|\varphi_i\rangle ds + f_i \int_0^1 \langle\varphi_i|\hat{H}_i^{\mathrm{PZ}}(s)|\varphi_i\rangle ds - E_{\mathrm{Hxc}}[\rho_i] \tag{S4}$$

where $\hat{H}_i^{\mathrm{PZ}}(s) = \hat{H}^{\mathrm{DFT}}(s) - \hat{v}_{\mathrm{Hxc}}^{\mathrm{DFT}}[s|\varphi_i(\mathbf{r})|^2]$. (Ref. S1 included an erroneous sum over i in the definition of this Hamiltonian.) Meanwhile, eq. 6 of Ref S2 defined KIPZ as

$$\Pi_i^{\mathrm{KIPZ}} = -\int_0^{f_i} \langle\varphi_i|\hat{H}^{\mathrm{DFT}}(s)|\varphi_i\rangle ds + f_i \int_0^1 \langle\varphi_i|\hat{H}_i^{\mathrm{PZ}}(s)|\varphi_i\rangle ds \tag{S5}$$

and in that same paper it was also stated that

$$\Pi_i^{\mathrm{KIPZ}} = \Pi_i^{\mathrm{KI}} - f_i E_{\mathrm{Hxc}}[n_i] \tag{S6}$$

One can prove that these three definitions are equivalent via the identity

$$\int_0^{f} \langle\varphi_i|v_{\mathrm{Hxc}}[sn_i]|\varphi_i\rangle ds = E_{\mathrm{Hxc}}[fn_i], \tag{S7}$$

from which it follows that

$$\begin{aligned}
&\int_0^{f_i} \langle\varphi_i|\hat{H}_i^{\mathrm{PZ}}(s)|\varphi_i\rangle ds \\
=&\int_0^{f_i} \langle\varphi_i|\hat{H}^{\mathrm{DFT}}(s)|\varphi_i\rangle ds - \int_0^{f_i} \langle\varphi_i|v_{\mathrm{Hxc}}[sn_i]|\varphi_i\rangle ds \\
=&\int_0^{f_i} \langle\varphi_i|\hat{H}^{\mathrm{DFT}}(s)|\varphi_i\rangle ds - E_{\mathrm{Hxc}}[f_i n_i]
\end{aligned} \tag{S8}$$

which proves that eqs. S4 and S5 are equivalent. Furthermore,

$$
\begin{aligned}
& -\int_0^{f_i} \langle\varphi_i|\hat{H}^{\mathrm{DFT}}(s)|\varphi_i\rangle ds + f_i \int_0^1 \langle\varphi_i|\hat{H}_i^{\mathrm{PZ}}(s)|\varphi_i\rangle ds \\
=& -\int_0^{f_i} \langle\varphi_i|\hat{H}^{\mathrm{DFT}}(s)|\varphi_i\rangle ds + f_i \int_0^1 \langle\varphi_i|\hat{H}_i^{\mathrm{DFT}}(s)|\varphi_i\rangle ds - f_i \int_0^1 \langle\varphi_i|v_{\mathrm{Hxc}}[sn_i]|\varphi_i\rangle ds \\
=& \Pi_i^{\mathrm{KI}} - f_i E_{\mathrm{Hxc}}[n_i]
\end{aligned}
\tag{S9}
$$

and thus eqs. S5 and S6 are equivalent.

In the unscreened case, the KIPZ functional as defined above is equivalent to the KI correction applied to an unscreened PZ base functional (i.e. KI@PZ). However, in the general case when screening is accounted for, KIPZ and KI@PZ are not equivalent. Instead, the PZ corrections incorporated within the KIPZ functional each inherit their own screening coefficient from the generalized PWL condition. This is desirable, because scaling down the PZ correction has been shown to improve energetics and thermochemistry,[S3–S5] but it would be interesting to explore alternative prescriptions for the scaling of the PZ correction that are decoupled from the generalized PWL condition.

S3 Details of the Koopmans workflows

This appendix contains a detailed breakdown of the two key Koopmans workflows: one for calculations where the screening parameters are calculated via finite differences (Figure S1), and the other via DFPT (Figure S2)

S3.1 The finite-difference workflow

In this workflow, we calculate screening parameters via the method described in Section 2.3 of the main text.

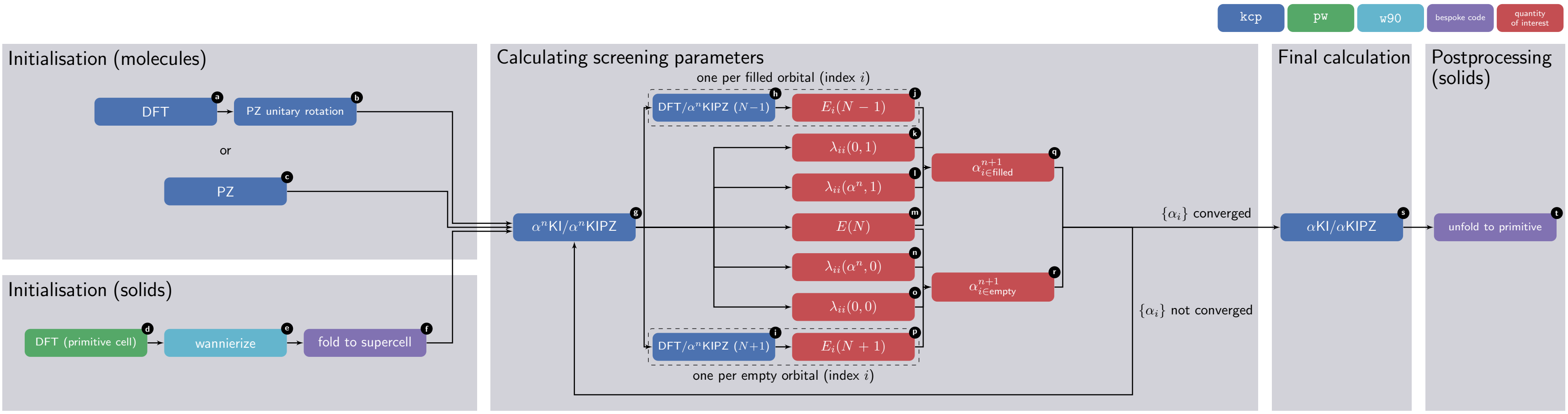

Figure S1: The finite-difference workflow. Individual nodes represent a calculation performed with QUANTUM ESPRESSO, except for the red nodes, which represent key quantities of interest that we extract from a preceding calculation. Individual nodes are explained more fully in the text.

S3.1.1 Initialization

The first step in this workflow is the initialization of the density and the variational orbitals. Depending on the system and functional in question, this can look quite different. For molecules, one can start with a DFT calculation to obtain the ground-state Kohn-Sham eigenvalues (node a in Figure S1). This is typical of KI calculations, which share the same ground-state density as the base DFT functional, in which case the final density has already been determined by this very first calculation. Note, however, that a unitary rotation of the occupied Kohn-Sham orbital densities leaves the total density (and therefore the total energy) unchanged, which means that the variational orbitals from a DFT calculation are not uniquely defined. In order to resolve this issue, one then performs a unitary rotation of the occupied Kohn-Sham variational orbitals to minimize the PZ energy (node b). This gives us a unique set of variational orbitals, while leaving the total density unchanged, and fulfils the definition of the KI functional as the $\gamma \rightarrow 0$ limit of the "KIγPZ" functional (as introduced in Section 2.4.3 in the main text). If one is using the KIPZ functional, it is better to initialize the density and the variational orbitals by performing a full PZ calculation (node c). In contrast to the previous KI approach where the DFT and KI ground-state densities match, the KIPZ and PZ ground-state densities and variational orbitals are similar but not identical, so the PZ solution serves as a suitable initial guess for a KIPZ calculation. Note that all of the above calculations are performed with the Γ-point-only `kcp.x` code.

For solids, the approach for initializing the density and variational orbitals is very different. Here, we take advantage of the periodicity of the lattice by initializing the variational orbitals using maximally localized Wannier functions (MLWFs). This approach is justified by the Wannier-like character of the true minimizing orbitals.[S6] Practically, the Wannierization procedure involves a DFT calculation with the `pw.x` code (node d), followed by a Wannierization procedure using the `Wannier90` and `pw2wannier90.x` codes (node e).

There are two important points when it comes to the Wannierization. The first is that the occupied and the empty manifolds must be Wannierized separately. This guarantees

that the occupancy matrix is diagonal in the basis of variational orbitals, as required by Koopmans functionals (see Section 2.5 of the main text). The second important point is that mixing bands that are far apart in energy-space is generally detrimental to the Koopmans results. To avoid this, each block of bands that are well-separated in energy-space are Wannierized separately, preventing inter-block mixing during the Wannierization procedure. This is a similar but cruder approach to the so-called dually-localized Wannier functions, where the Wannier functions minimize a localization criteria that is a mix of spatial and energy localization.[S7]

The one final task is to map the Wannier functions in the k-sampled primitive cell to the equivalent Γ-point-only supercell in a format readable by the `kcp.x` code that will handle the subsequent calculation of the screening parameters (node f). In this procedure the supercell dimensions match those of the k-grid used during the initialization.

S3.1.2 Calculating the screening parameters

Having initialized the density and the variational orbitals, the next task to perform is the calculation of the screening parameters. To this end, let us restate eqs. 10 and 11 from the main text, with which we calculate these parameters:

$$\alpha_i^{n+1} = \alpha_i^n \frac{\Delta E_i^{\text{Koopmans}} - \lambda_{ii}(0,1)}{\lambda_{ii}(\alpha_i^n, 1) - \lambda_{ii}(0,1)}; \qquad \Delta E_i^{\text{Koopmans}} = E^{\text{Koopmans}}(N) - E_i^{\text{Koopmans}}(N-1) \tag{S10}$$

for occupied orbitals and

$$\alpha_i^{n+1} = \alpha_i^n \frac{\Delta E_i^{\text{Koopmans}} - \lambda_{ii}(0,0)}{\lambda_{ii}(\alpha_i^n, 0) - \lambda_{ii}(0,0)}; \qquad \Delta E_i^{\text{Koopmans}} = E_i^{\text{Koopmans}}(N+1) - E^{\text{Koopmans}}(N) \tag{S11}$$

for empty orbitals. In order to calculate these screening parameters, we therefore require three calculations. The first calculation is a KI or KIPZ calculation with using a trial screening parameter α^0 (node g). This gives us access to the energy of the N-electron system

$E(N)$ (node m) as well as all of the requisite expectation values Koopmans Hamiltonian on the variational orbitals $\lambda_{ii}(\alpha, f)$ (nodes k, l, n, and o). The second and third calculations (nodes h and i) are calculations on the $N \pm 1$-electron systems, where orbital i is frozen and its occupancy is fixed to 0 (in the case of occupied orbitals) or 1 (empty orbitals). These calculations yield the total energies $E_i(N \pm 1)$ (nodes j and p). For these calculations in particular, ensuring that there is no spurious interactions between images is crucial (because now we have a charged defect in our system). This requires the use of both a sufficiently large supercell and a correction scheme such as Gygi-Baldereschi.[S8] Note that since KI yields the same total energies as the base functional, these two calculations can be performed at the DFT level when performing the KI workflow.

Having performed these three calculations (nodes g-i) and extracted all of the requisite information (nodes j-p), we can then calculate the screening parameters (nodes q and r) according to the above equations. If the screening parameters are converged, we can then proceed to the final calculation; if not, the process is repeated.

We note that this iterative procedure almost universally converges very quickly. Indeed, for the KI functional and with occupied orbitals, it is guaranteed to converge instantly. This is because, as mentioned earlier, ΔE_i is independent of α, as are occupied variational orbitals, and consequently $\lambda_{ii}^{\mathrm{KI}}(\alpha, 1)$ is linear in α. This is not the case for empty orbitals for the KI functional (for which $\lambda_{ii}^{\mathrm{KI}}(\alpha, 0)$ is not strictly linear) or for the KIPZ functional (where additionally ΔE_i is dependent on α). Even for these functionals, the screening parameters tend to converge in a few iterations.

S3.1.3 The final calculation and postprocessing for solids

We now perform a KI or KIPZ calculation with the finalized set of screening parameters (node s). For a molecular system we are now done: the KI/KIPZ calculation yields a Hamiltonian in the basis of variational orbitals which we diagonalize to extract the quasiparticle energies.

However, for a calculation on a periodic system one final step is required. This is because

all of the preceding `kcp.x` calculations have been performed in a Γ-point-only supercell. In order to extract the band structure, we must now unfold the band structure by taking advantage of the MLWF basis, as described in Ref. S6. This step is performed within python by the `koopmans` workflow manager itself. One trick that we can perform at this stage is "smooth interpolation". In the supercell, our Hamiltonian in the basis of Wannier functions is given by

$$h_{mn}^{\mathrm{DFT}}(\mathbf{R}) + v_{mn}^{\mathrm{Koopmans}}(\mathbf{R}) \tag{S12}$$

The Koopmans potential is very smooth and slowly-varying in **k**-space, applying an almost-constant shift to the Kohn-Sham DFT bands. Consequently, the dominant contribution to the dispersion of the bands comes from the DFT Hamiltonian, and it makes sense to construct the **k**-indexed Hamitonian as

$$h_{mn}(\mathbf{k}) = \sum_{\mathbf{R}'} e^{i\mathbf{k}\cdot\mathbf{R}'} h_{mn}^{\mathrm{DFT}}(\mathbf{R}') + \sum_{\mathbf{R}} e^{i\mathbf{k}\cdot\mathbf{R}} v_{mn}^{\mathrm{Koopmans}}(\mathbf{R}) \tag{S13}$$

where now $\{\mathbf{R}'\}$ corresponds to a much larger supercell or, equivalently, a much denser **k**-point grid. The advantage of this strategy is that it improves the interpolation of the band structure at very little computational cost. Suppose we perform a smooth interpolation with a grid twice as fine as the default grid. The only additional computational cost in this instance is having to generate the DFT Hamiltonian in the Wannier basis for this finer grid (i.e. we repeat nodes d and e). This only represents a small fraction of the total workflow, and includes only DFT and not ODDFT calculations, so it only fractionally increases the total computational cost. Contrast this with the alternative, where one could perform the entire calculation with a grid twice as fine. This would require us to perform (among other things) calculations on a supercell containing eight times as many atoms, drastically increasing the computational cost of the workflow as a whole. For more details on the smooth interpolation procedure, refer to Ref. S6.

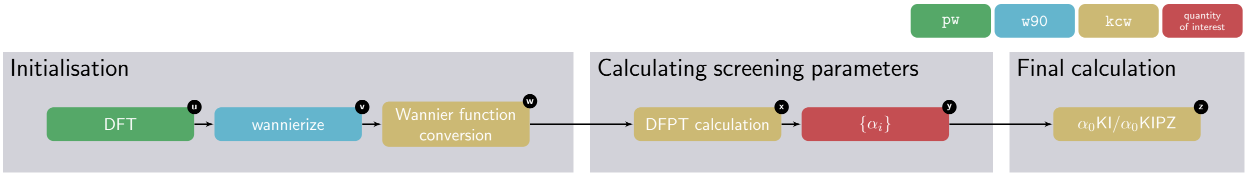

Figure S2: The DFPT workflow. Individual nodes are explained in the text.

S3.2 The DFPT workflow

The DFPT workflow is depicted in Figure S2. It is simpler than the finite-difference procedure, because orbital relaxation is not implemented, and instead Wannier functions are used as approximations to the true variational orbitals. This means that only the KI and pKIPZ functionals can be used in this scheme, means that the screening parameters do not need to be calculated self-consistently, and makes the Koopmans functional effectively a post-processing step on top of a DFT calculation.

S3.2.1 Initialization

The initialization procedure for the DFPT workflow is very similar to that for solids in the finite-difference workflow. A primitive cell calculation `pw.x` calculation (node u) is followed by a Wannierization procedure in order to define the density and the variational orbitals (node v). The one difference is that now, instead of using `kcw.x` to map these Wannier functions to a supercell that is readable by `kcp.x`, we use `kcw.x` to convert the Wannier functions to more convenient format for subsequent calculations (node w). Note that `kcw.x` does not map to a supercell because this workflow operates entirely within the primitive cell with k-point sampling.

S3.2.2 Calculating the screening parameters

DFPT calculations evaluating eq. 21 (from the main text) are then performed by a single `kcw.x` run (node x). These calculations yield the screening parameters (node y).

S3.2.3 The final calculation and postprocessing for solids

Having calculated the screening parameters, the Koopmans Hamiltonian is constructed in the basis of Wannier functions and then diagonalized, resulting in the full band structure of the system at hand (node z). Like the previous calculations, this is performed using `kcw.x`.

S4 Example input and output files

Below are example `koopmans` input and output files for ozone, silicon, and zinc oxide. All of these files can also be found on Materials Cloud at 10.24435/materialscloud:9w-sp.

S4.1 Ozone

An example minimal input file for ozone is as follows

```
{
  "workflow": {
    "functional": "ki",
    "method": "dscf",
    "init_orbitals": "kohn-sham",
    "n_max_sc_steps": 5,
    "keep_tmpdirs": false,
    "pseudo_library": "sg15"
  },
  "atoms": {
    "cell_parameters": {
      "periodic": false,
      "vectors": [[14.1738, 0.0, 0.0],
                  [0.0, 12.0, 0.0],
                  [0.0, 0.0, 12.66]],
      "units": "angstrom"
    },
    "atomic_positions": {
      "positions": [["O", 7.0869, 6.0, 5.89],
                    ["O", 8.1738, 6.0, 6.55],
                    ["O", 6.0, 6.0, 6.55]],
      "units": "angstrom"
    }
  },
```

```
25    "calculator_parameters": {
26      "ecutwfc": 65.0,
27      "nbnd": 10
28    }
29  }
```

This input file contains several blocks. The `workflow` block allows the user to specify the details of the workflow. Here we can see we are performing a KI calculation (line 3) calculating the screening parameters via the finite-difference procedure (line 4), and using the Kohn-Sham orbitals to initialize our variational orbitals (line 5; this is common practice for molecules). The `atoms` block (lines 10-23) contains standard keywords specifying the system configuration, such as the `cell_parameters` and `atomic_positions`. These mirror the equivalent blocks in `Quantum ESPRESSO` input files (albeit in JSON format). Finally, the `calculator_parameters` block allows the user to specify settings specific to a particular code (e.g. a `w90` subblock for specifying `Wannier90` settings). In this instance we are providing a particular energy cutoff (line 26) and specifying the total number of orbitals to compute (line 27).

The output of `koopmans ozone.json`, which prompts a sequence of `Quantum ESPRESSO` calculations necessary to initialize the density and variational orbitals (lines 16-22), calculate the screening parameters (lines 24-68), and run a final KI calculation (lines 85-87).

```
1     _
2    | | _____   ___  _ __  _ __ ___   __ _ _ __  ___
3    | |/ / _ \ / _ \| '_ \| '_ ` _ \ / _` | '_ \/ __|
4    |   < (_) | (_) | |_) | | | | | | (_| | | | \__ \
5    |_|\_\___/ \___/| .__/|_| |_| |_|\__,_|_| |_|___/
6                    |_|
7
8    Koopmans spectral functional calculations with Quantum ESPRESSO


       ...

16     Initialization of density and variational orbitals
17     ==================================================
18      Running init/dft_init_nspin1... done
19      Running init/dft_init_nspin2_dummy... done
20      Running init/dft_init_nspin2... done
21      Overwriting the variational orbitals with Kohn-Sham orbitals
```

```
 Copying the spin-up variational orbitals over to the spin-down channel

Calculating screening parameters
================================

SC iteration 1
--------------
 Running calc_alpha/iteration_1/ki... done

Orbital 1
---------
 Running calc_alpha/iteration_1/orbital_1/dft_n-1... done


  ...
Orbital 10
----------
 Running calc_alpha/iteration_1/orbital_10/pz_print... done
 Running calc_alpha/iteration_1/orbital_10/dft_n+1_dummy... done
 Running calc_alpha/iteration_1/orbital_10/dft_n+1... done


  ...
SC iteration 2
--------------
 Running calc_alpha/iteration_2/ki_nspin1_dummy... done
 Running calc_alpha/iteration_2/ki_nspin1... done
 Running calc_alpha/iteration_2/ki_nspin2_dummy... done
 Running calc_alpha/iteration_2/ki_nspin2... done

Orbital 10
----------
 Running calc_alpha/iteration_2/orbital_10/pz_print... done
 Running calc_alpha/iteration_2/orbital_10/dft_n+1_dummy... done
 Running calc_alpha/iteration_2/orbital_10/dft_n+1... done

 alpha
          1         2         3        4   ...        7       8         9        10
 0  0.600000  0.600000  0.600000  0.60000  ...  0.600000  0.6000  0.600000  0.600000
 1  0.655689  0.727571  0.783859  0.66386  ...  0.729888  0.7419  0.779264  0.717389
 2  0.655689  0.727571  0.783859  0.66386  ...  0.729888  0.7419  0.779264  0.717389


  ...
 Screening parameters have been converged

Final KI calculation
====================
 Running final/ki_final... done

Workflow complete
```

S4.2 Silicon

A typical input file for silicon is similar to that of ozone, but with some tweaked workflow settings, a `cell_parameters` block that denotes that this system is periodic, and some additional Wannierization settings:

```
{
  "workflow": {
    "functional": "ki",
    "base_functional": "pbe",
    "method": "dscf",
    "init_orbitals": "mlwfs",
    "alpha_guess": 0.1,
    "eps_inf": 13.02,
    "orbital_groups_self_hartree_tol": 0.1,
    "pseudo_library": "sg15",
    "mp_correction": false
  },
  "atoms": {
    "atomic_positions": {
      "units": "crystal",
      "positions": [["Si", 0.00, 0.00, 0.00],
                    ["Si", 0.25, 0.25, 0.25]]
    },
    "cell_parameters": {
      "periodic": true,
      "ibrav": 2,
      "celldms": {"1": 10.2622}
    }
  },
  "kpoints": {
    "grid": [4, 4, 4]
  },
  "calculator_parameters": {
    "ecutwfc": 60.0,
    "pw": {
      "nbnd": 20
    },
    "w90": {
      "projections": [
        [{"fsite": [0.25, 0.25, 0.25], "ang_mtm": "sp3"}],
        [{"fsite": [0.25, 0.25, 0.25], "ang_mtm": "sp3"}]],
      "dis_froz_max": 11.0,
      "dis_win_max": 16.5
    },
    "ui": {
      "smooth_int_factor": 2
    }
  }
}
```

For a full explanation of the meaning of the `Wannier90` keywords we refer the reader to the `Wannier90` documentation.

Running `koopmans si.json` generates the following output. This command prompts a sequence of `Quantum ESPRESSO` calculations necessary to initialize the density and variational orbitals using Wannier functions (lines 20-29), fold these k-resolved functions to the equivalent Γ-only supercell (lines 31-36) calculate the screening parameters (lines 38-73), run a final KI calculation (lines 75-77), and finally perform a second DFT Wannierization on a finer k-grid to produce a smoothly interpolated band structure (lines 80-97).

```
1   _
2  | | _____   ___  _ __  _ __ ___   __ _ _ __  ___
3  | |/ / _ \ / _ \| '_ \| '_ ` _ \ / _` | '_ \/ __|
4  |   < (_) | (_) | |_) | | | | | | (_| | | | \__ \
5  |_|\_\___/ \___/| .__/|_| |_| |_|\__,_|_| |_|___/
6                  |_|
7
8  Koopmans spectral functional calculations with Quantum ESPRESSO


     ...

17  Initialization of density and variational orbitals
18  ==================================================
19
20   Wannierization
21   ==============
22    Running wannier/scf... done
23    Running wannier/nscf... done
24    Running wannier/block_1/wann_preproc... done
25    Running wannier/block_1/pw2wan... done
26    Running wannier/block_1/wann... done
27    Running wannier/block_2/wann_preproc... done
28    Running wannier/block_2/pw2wan... done
29    Running wannier/block_2/wann... done
30
31   Folding to supercell
32   --------------------
33    Running block_1/w2kcp... done
34    Running block_2/w2kcp... done
35   Running init/dft_dummy... done
36   Running init/dft_init... done
37
38  Calculating screening parameters
39  ================================
40   Running calc_alpha/ki... done
41
42  Orbitals 1-255
```

```
  --------------
   Skipping; will use the screening parameter of an equivalent orbital

  Orbital 256
  -----------
   Running calc_alpha/orbital_256/dft_n-1... done

  Orbital 257
  -----------
   Running calc_alpha/orbital_257/pz_print... done
   Running calc_alpha/orbital_257/dft_n+1_dummy... done
   Running calc_alpha/orbital_257/dft_n+1... done

  Orbitals 258-512
  ----------------
   Skipping; will use the screening parameter of an equivalent orbital

   alpha
             1         2         3    ...       510       511       512
   0  0.100000  0.100000  0.100000  ...  0.100000  0.100000  0.100000
   1  0.104648  0.104648  0.104648  ...  0.047709  0.047709  0.047709

   [2 rows x 512 columns]

   Delta E_i - epsilon_i (eV)
             1         2         3    ...       510       511       512
   0 -0.023309 -0.023309 -0.023309  ... -0.139624 -0.139624 -0.139624

   [1 rows x 512 columns]

   Screening parameters have been determined but are not necessarily converged

  Final KI calculation
  ====================
   Running final/ki_final... done


  Postprocessing
  ==============

    Wannierization
    ==============
     Running wannier/scf... done
     Running wannier/nscf... done
     Running wannier/block_1/wann_preproc... done
     Running wannier/block_1/pw2wan... done
     Running wannier/block_1/wann... done
     Running wannier/block_2/wann_preproc... done
     Running wannier/block_2/pw2wan... done
     Running wannier/block_2/wann... done
     Running wannier/bands... done
UserWarning: Some of the pseudopotentials do not have PP_PSWFC blocks, which means a
↪  projected DOS calculation is not possible. Skipping...
    Running occ/ki... done
```

```
    Running emp/ki... done
UserWarning: The DOS will not be plotted, because the Brillouin zone is too poorly
↪  sampled for the specified value of smearing. In order to generate a DOS, increase the
↪  k-point density ("kpath_density" in the "setup" "k_points" subblock) and/or the
↪  smearing ("degauss" in the "plot" block)

 Workflow complete
```

S4.3 Zinc oxide

The input file is as follows:

```
{
    "workflow": {
        "task": "singlepoint",
        "functional": "ki",
        "base_functional": "lda",
        "method": "dfpt",
        "init_orbitals": "mlwfs",
        "calculate_alpha" : true,
        "pseudo_library": "pseudo_dojo_standard",
        "gb_correction" : true,
        "eps_inf": 5.3,
        "from_scratch": true,
        "npool": 4,
        "dfpt_coarse_grid": [2, 2, 2],
        "orbital_groups_spread_tol": 0.0005
    },
    "atoms": {
       "cell_parameters": {
         "periodic": true,
         "ibrav": 4,
         "celldms": {"1": 6.14057, "3": 1.60204}
        },
        "atomic_positions": {
            "units": "crystal",
            "positions": [
                ["Zn", 0.33330, 0.66670, 0.50000 ],
                ["Zn", 0.66670, 0.33330, 0.00000 ],
                ["O" , 0.33330, 0.66670, 0.11725 ],
                ["O" , 0.66670, 0.33330, 0.61725 ]
            ]
        }
    },
    "kpoints": {
        "grid": [
            4,
            4,
            4
        ],
        "path": "ALMGAHK"
    },
    "calculator_parameters": {
        "ecutwfc": 50.0,
        "pw": {
            "system": {
                "nbnd": 52
            }
        },
        "w90": {
            "conv_tol": 1e-8,
```

```
50            "projections": [
51                [{"site": "Zn", "ang_mtm": "l=0"}],
52                [{"site": "Zn", "ang_mtm": "l=1"}],
53                [{"site": "O", "ang_mtm": "l=0"}],
54                [{"site": "Zn", "ang_mtm": "l=2"},
55                 {"site": "O", "ang_mtm": "l=1"}],
56                [{"site": "Zn", "ang_mtm": "l=0"}]
57            ],
58            "dis_froz_max": 14.5,
59            "dis_win_max": 17.0
60        }
61    },
62    "plotting": {
63        "Emin": -10,
64        "Emax": 10
65    }
66 }
```

Here we can see the selection of DFPT for calculating screening parameters (line 6), the choice of MLWFs as the variational orbitals (line 7), and a criterion for grouping variational orbitals together based on their spreads (line 15). We also have specified a coarse $2 \times 2 \times 2$ k-point grid on which to calculate the screening parameters (line 14) relative to the $4 \times 4 \times 4$ grid upon which we construct the Hamiltonian (specified elsewhere in the output file). Again, it is worth stressing that these calculations are not fully converged.

The output of `koopmans zno.json` is below. This command prompts a sequence of `Quantum ESPRESSO` calculations which, using a coarse grid, calculates the density and variational orbitals using Wannier functions (lines 20-40) and then calculates the screening parameters using DFPT (lines 46-57). Then, on a regular grid, it repeats a Wannierization (62-82) and then constructs and diagonalizes the Koopmans Hamiltonian (lines 88-90), using the screening parameters calculated on the coarse grid.

```
1   _
2  | | _____   ___  _ __  _ __ ___   __ _ _ __  ___
3  | |/ / _ \ / _ \| '_ \| '_ ` _ \ / _` | '_ \/ __|
4  |   < (_) | (_) | |_) | | | | | | (_| | | | \__ \
5  |_|\_\___/ \___/| .__/|_| |_| |_|\__,_|_| |_|___/
6                  |_|
7
8  Koopmans spectral functional calculations with Quantum ESPRESSO
```

...

```
Coarse grid calculations
========================

  Wannierization
  ==============
   Running wannier/scf... done
   Running wannier/nscf... done
   Running wannier/block_1/wann_preproc... done
   Running wannier/block_1/pw2wan... done
   Running wannier/block_1/wann... done
   Running wannier/block_2/wann_preproc... done
   Running wannier/block_2/pw2wan... done
   Running wannier/block_2/wann... done
   Running wannier/block_3/wann_preproc... done
   Running wannier/block_3/pw2wan... done
   Running wannier/block_3/wann... done
   Running wannier/block_4/wann_preproc... done
   Running wannier/block_4/pw2wan... done
   Running wannier/block_4/wann... done
   Running wannier/block_5/wann_preproc... done
   Running wannier/block_5/pw2wan... done
   Running wannier/block_5/wann... done
   Running wannier/bands... done
   Running pdos/projwfc... done

 Conversion to Koopmans format
 -----------------------------
  Running wannier/kc... done

 Calculation of screening parameters
 ===================================
  Running screening/band_2/kc... done
  Running screening/band_6/kc... done
  Running screening/band_8/kc... done
  Running screening/band_10/kc... done
  Running screening/band_16/kc... done
  Running screening/band_18/kc... done
  Running screening/band_20/kc... done
  Running screening/band_24/kc... done
  Running screening/band_26/kc... done
  Running screening/band_27/kc... done

Regular grid calculations
=========================

 Wannierization
 ==============
  Running wannier/scf... done
  Running wannier/nscf... done
  Running wannier/block_1/wann_preproc... done
  Running wannier/block_1/pw2wan... done
  Running wannier/block_1/wann... done
  Running wannier/block_2/wann_preproc... done
  Running wannier/block_2/pw2wan... done
```

```
    Running wannier/block_2/wann... done
    Running wannier/block_3/wann_preproc... done
    Running wannier/block_3/pw2wan... done
    Running wannier/block_3/wann... done
    Running wannier/block_4/wann_preproc... done
    Running wannier/block_4/pw2wan... done
    Running wannier/block_4/wann... done
    Running wannier/block_5/wann_preproc... done
    Running wannier/block_5/pw2wan... done
    Running wannier/block_5/wann... done
    Running wannier/bands... done
    Running pdos/projwfc... done

  Conversion to Koopmans format
  -----------------------------
   Running wannier/kc... done

  Construction of the Hamiltonian
  ===============================
   Running hamiltonian/kc... done

 Workflow complete
```

S5 Details of the koopmans package

S5.1 Code structure

koopmans is built on top of the ASE python package (the Atomic Simulation Environment).[S9] Under the hood, it defines various Workflow classes, which look like

```
class Workflow:
   parameters: Dict[str, Any]
   calculations: List[Calculator]
   ...
```

where the parameters attribute is a dictionary that stores the workflow parameters as specified in the input file, and calculations is a list of the calculations in the workflow. The individual entries in the calculations list correspond to Calculator objects:

```
class Calculator(ASE_Calculator):
   atoms: Atoms
   parameters: Dict[str, Any]
   results: Dict[str, Any]
   ...
```

which are subclasses of corresponding classes defined by ASE. ASE provides the calculator with the functionality to read and write input and output files (among many other things).

A Calculator object has — among others — an atoms attribute that stores the details of the atoms and the simulation cell. The atoms attribute is itself an instance of the Atoms class from ASE. We note that this hierarchy (namely, that the atoms object is an attribute of a Calculator, and not the other way around) is the reverse of the philosophy of ASE, where Atoms objects are the principal object, and they may or may not have an associated calc attribute.

In addition to an atoms attribute, Calculator objects also have a parameters attribute where calculator-specific settings are stored, as well as a results attribute, where the results of the calculation are stored — just like in ASE.

S5.2 Scriptability

Because koopmans is written in python, integrating it within a script is straightforward. For example, here is a script that runs the ozone calculation from Section 4.1:

```python
from ase import build
from koopmans.workflows import SinglepointWorkflow

# Create an Atoms object
atoms = build.molecule('O3', vacuum=5.0)

# Create a koopmans Workflow object
workflow = SinglepointWorkflow(atoms=atoms, ecutwfc = 65.0, nbnd = 10)

# Run the workflow
workflow.run()

# Fetch the IP and EA
results = workflow.calculations[-1].results
ip = -results['homo_energy']
ea = -results['lumo_energy']

# Print the IP and EA to screen
print(f' IP = {ip:.2f} eV')
print(f' EA = {ea:.2f} eV')
```

Of course, printing the IP and EA to screen is of limited value — in reality at this stage the user would then generate plots, or feed these results to another code.

Often a user will want to run workflows and analyse data separately — for example, they might run their workflow on remote high performance computing resources, and then, days later, analyse the results on their laptop. To permit this, `koopmans` generates a `.kwf` file when a workflow is run. This file can be loaded into python in order to recover the `Workflow` python object. For example, we could perform exactly the same analysis on our previous ozone calculation by replacing lines 1-11 with

```
from koopmans import io
workflow = io.read('ozone.kwf')
```

where `ozone.kwf` has been generated by some previously completed `koopmans` calculation.

In the above, we used the `SinglepointWorkflow` for running a Koopmans workflow from start to finish. `koopmans` implements several other workflows that automate tasks that are useful when performing Koopmans calculations, such as convergence testing, standalone Wannierization, and DFT calculations.

S5.3 Code quality and testing

`koopmans` contains an extensive test suite implemented with `pytest`.[S10] It also has typing annotations which allow it to be statically typechecked using `mypy`.

References

(S1) Borghi, G.; Ferretti, A.; Nguyen, N. L.; Dabo, I.; Marzari, N. Koopmans-Compliant Functionals and Their Performance against Reference Molecular Data. *Phys. Rev. B* **2014**, *90*, 075135.

(S2) Nguyen, N. L.; Colonna, N.; Ferretti, A.; Marzari, N. Koopmans-Compliant Spectral Functionals for Extended Systems. *Phys. Rev. X* **2018**, *8*, 021051.

(S3) Vydrov, O. A.; Scuseria, G. E.; Perdew, J. P.; Ruzsinszky, A.; Csonka, G. I. Scaling down the Perdew-Zunger Self-Interaction Correction in Many-Electron Regions. *J. Chem. Phys.* **2006**, *124*, 094108.

(S4) Bylaska, E. J.; Tsemekhman, K.; Gao, F. New Development of Self-Interaction Corrected DFT for Extended Systems Applied to the Calculation of Native Defects in 3C–SiC. *Phys. Scr.* **2006**, *2006*, 86.

(S5) Valdés, Á.; Brillet, J.; Grätzel, M.; Gudmundsdóttir, H.; Hansen, H. A.; Jónsson, H.; Klüpfel, P.; Kroes, G.-J.; Formal, F. L.; Man, I. C.; Martins, R. S.; Nørskov, J. K.; Rossmeisl, J.; Sivula, K.; Vojvodic, A.; Zäch, M. Solar Hydrogen Production with Semiconductor Metal Oxides: New Directions in Experiment and Theory. *Phys. Chem. Chem. Phys.* **2011**, *14*, 49–70.

(S6) De Gennaro, R.; Colonna, N.; Linscott, E.; Marzari, N. Bloch's Theorem in Orbital-Density-Dependent Functionals: Band Structures from Koopmans Spectral Functionals. *Phys. Rev. B* **2022**, *106*, 035106.

(S7) Mahler, A.; Williams, J. Z.; Su, N. Q.; Yang, W. Wannier Functions Dually Localized in Space and Energy. `arXiv:2201.07751`, 2022.

(S8) Gygi, F.; Baldereschi, A. Self-Consistent Hartree-Fock and Screened-Exchange Calculations in Solids: Application to Silicon. *Phys. Rev. B* **1986**, *34*, 4405–4408.

(S9) Larsen, A. H.; Mortensen, J. J.; Blomqvist, J.; Castelli, I. E.; Christensen, R.; Dułak, M.; Friis, J.; Groves, M. N.; Hammer, B.; Hargus, C.; Hermes, E. D.; Jennings, P. C.; Bjerre Jensen, P.; Kermode, J.; Kitchin, J. R.; Leonhard Kolsbjerg, E.; Kubal, J.; Kaasbjerg, K.; Lysgaard, S.; Maronsson, J. B.; Maxson, T.; Olsen, T.; Pastewka, L.; Peterson, A.; Rostgaard, C.; Schiøtz, J.; Schütt, O.; Strange, M.; Thygesen, K. S.; Vegge, T.; Vilhelmsen, L.; Walter, M.; Zeng, Z.; Jacobsen, K. W. The Atomic Simulation Environment—a Python Library for Working with Atoms. *J. Phys. Condens. Matter* **2017**, *29*, 273002.

(S10) Krekel, H.; Oliveira, B.; Pfannschmidt, R.; Bruynooghe, F.; Laugher, B.; Bruhin, F. Pytest 7.1. `https://github.com/pytest-dev/pytest`, 2004; Accessed 05/06/2022.
